# Whole Exome Sequencing of 23 Multigeneration Idiopathic Scoliosis Families Reveals Enrichments in Cytoskeletal Variants, Suggests Highly Polygenic Disease

**DOI:** 10.3390/genes12060922

**Published:** 2021-06-16

**Authors:** Elizabeth A. Terhune, Cambria I. Wethey, Melissa T. Cuevas, Anna M. Monley, Erin E. Baschal, Morgan R. Bland, Robin Baschal, G. Devon Trahan, Matthew R. G. Taylor, Kenneth L. Jones, Nancy Hadley Miller

**Affiliations:** 1 Department of Orthopedics, University of Colorado Anschutz Medical Campus, Aurora, CO 80045, USA; elizabeth.a.terhune@cuanschutz.edu (E.A.T.); cambria.wethey@cuanschutz.edu (C.I.W.); Melissa.cuevas@cuanschutz.edu (M.T.C.); anna.monley@cuanschutz.edu (A.M.M.); erin.baschal@cuanschutz.edu (E.E.B.); morganrbland@gmail.com (M.R.B.); robin.baschal@cuanschutz.edu (R.B.); 2 Musculoskeletal Research Center, Children’s Hospital Colorado, Aurora, CO 80045, USA; 3 Department of Pediatrics, University of Colorado Anschutz Medical Campus, Aurora, CO 80045, USA; devon.trahan@cuanschutz.edu (G.D.T.); ken.jones@ouhsc.edu (K.L.J.); 4 Department of Medicine, Adult Medical Genetics Program, University of Colorado Anschutz Medical Campus, Aurora, CO 80045, USA; matthew.taylor@cuanschutz.edu; 5 Department of Cell Biology, University of Oklahoma Health Science Center, Oklahoma City, OK 73104, USA

**Keywords:** idiopathic scoliosis, exome sequencing, spine, polygenic, variants, musculoskeletal disease, cytoskeleton, extracellular matrix

## Abstract

Adolescent idiopathic scoliosis (AIS) is a lateral spinal curvature >10° with rotation that affects 2–3% of healthy children across populations. AIS is known to have a significant genetic component, and despite a handful of risk loci identified in unrelated individuals by GWAS and next-generation sequencing methods, the underlying etiology of the condition remains largely unknown. In this study, we performed exome sequencing of affected individuals within 23 multigenerational families, with the hypothesis that the occurrence of rare, low frequency, disease-causing variants will co-occur in distantly related, affected individuals. Bioinformatic filtering of uncommon, potentially damaging variants shared by all sequenced family members revealed 1448 variants in 1160 genes across the 23 families, with 132 genes shared by two or more families. Ten genes were shared by >4 families, and no genes were shared by all. Gene enrichment analysis showed an enrichment of variants in cytoskeletal and extracellular matrix related processes. These data support a model that AIS is a highly polygenic disease, with few variant-containing genes shared between affected individuals across different family lineages. This work presents a novel resource for further exploration in familial AIS genetic research.

## 1. Introduction

Adolescent idiopathic scoliosis (AIS) is a structural lateral spinal curvature ≥10° that affects 2–3% of healthy children [1], with females at the greatest risk for severe progression [2,3,4]. In individuals with severe progressive curvatures, life-long problems of cosmetic deformity, respiratory compromise, back pain, and degenerative disease often arise and in many cases require surgical intervention, placing a significant economic burden upon our healthcare system [5]. AIS is known to be highly heritable, however, our knowledge of its etiology is severely limited, both in terms of the individuals at risk for curve initiation and those likely to experience curve progression. Understanding key risk variants or genetic pathways leading to AIS holds the potential to improve patient care by way of targeted clinical treatments or prognostics for detecting AIS risk or risk of curvature progression. 

Multiple studies support the genetic foundation of AIS, with sibling recurrence risks reported to be near 18%, and heritability estimates of approximately 87.5% [6,7,8]. To understand the genetics of AIS, traditional approaches including genome wide association studies (GWAS), exome sequencing, and familial linkage studies have been applied to multiple populations. GWAS of unrelated individuals with AIS have identified potential common risk alleles, notably those in or near *LBX1* [9,10,11,12,13,14,15,16,17,18,19,20,21,22,23] and *GPR126/ADGRG6* [23,24,25,26,27], which are the most well-replicated genetic findings to date across populations. Next-generation sequencing studies (i.e., whole exome sequencing) both within families and unrelated individuals with AIS have identified *rare* variants in extracellular matrix genes that may contribute to the AIS phenotype [28,29,30]. Recent genetic and functional studies have led to varied hypotheses of AIS etiology, including dysfunction within neuroinflammatory pathways [31,32], the cartilage matrisome [33], cilia, the cytoskeleton [34,35,36,37,38], or the vestibular system [39,40,41,42,43]. However, the inability thus far to relate specific genetic variants to the biology of AIS and the wide variation in which AIS presents are indicative of the complex heterogeneity of this disorder.

Disease susceptibility variants for complex diseases may collectively be common in the general population, but specific variants may be rare within families or affected individuals. Family studies present unique advantages over case-control studies, as they may reveal rare disease-associated variants enriched within the family that are amenable to targeted sequencing. Sequencing studies of multiple large families can reveal multiple variants within a gene or molecular pathway that contribute to the disease phenotype [44,45]. 

Within our laboratory, a pilot exome sequencing study of five AIS families identified an enrichment of damaging uncommon variants in cilia, extracellular matrix, and cytoskeletal genes, although no specific variants or genes were found to be present across all families [46]. 

This led us to propose the hypothesis that the development of AIS may be due to damaging variants within a specific set of pathways or molecular classes, rather than being driven by just a few select ‘AIS genes’. In this study, we expand upon our previous work and present exome sequencing of affected individuals from 23 AIS families, interpreted using gene enrichment analyses to identify overrepresented functional categories. We then investigate specific genes containing variants in multiple families via genotyping confirmation in additional affected and unaffected members of the family to assess how closely these variants track with the AIS phenotype.

## 2. Materials and Methods

**Figure 1:**
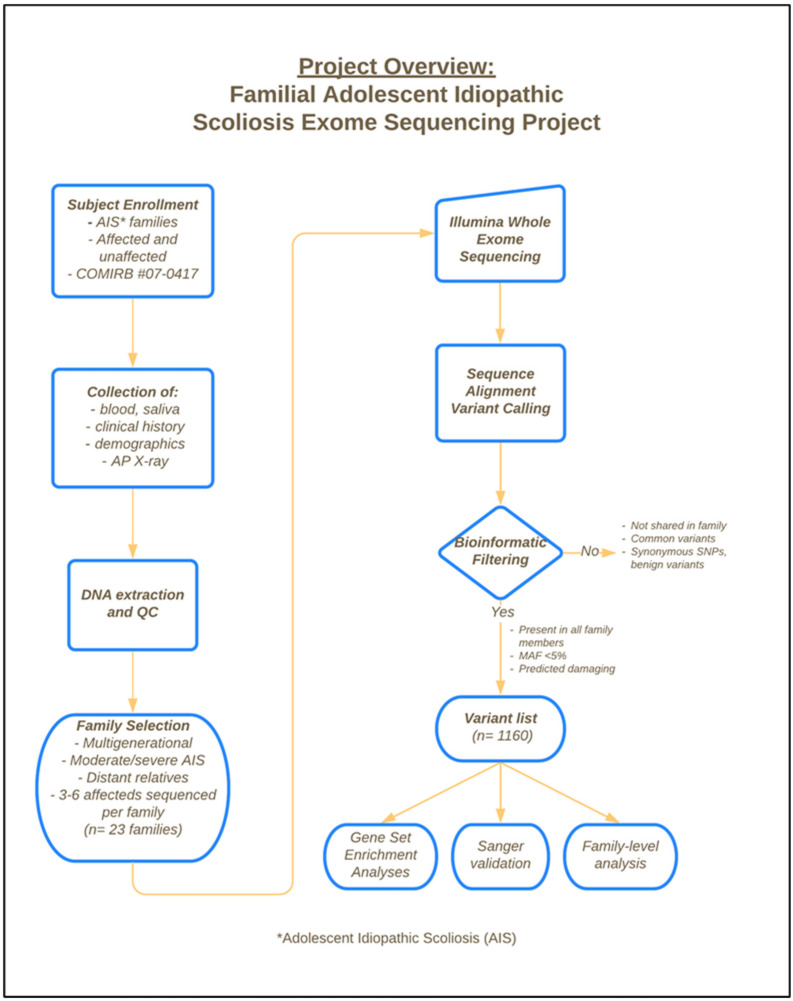
Project overview of subject enrollment, sample collection and extraction, exome sequencing, and bioinformatic filtering strategy.

An overview of the methodology for this study—including subject enrollment, sample collection, sequencing, and bioinformatic filtering strategies—are provided in Figure 1.

### 2.1. Subjects

Study subjects were enrolled as previously described [29,38,46]. Inclusion in the sequencing pool required a standing anteroposterior spinal radiograph showing ≥10° curvature by the Cobb method with pedicle rotation, and no evidence of congenital deformity or other co-existing genetic disorders [47,48,49]. 

Blood samples were collected from all study subjects as described previously [38]. DNA was then extracted from whole blood using standard phenol chloroform protocols or the QIAGEN Gentra PureGene Blood Kit. DNA quality was verified by Qubit (Invitrogen, Waltham, MA, USA), agarose gel electrophoresis and Nanodrop (Thermo Scientific, Waltham, MA, USA).

### 2.2. Family Selection for Exome Sequencing

Twenty-three large families were selected through a tiered process wherein each pedigree was reviewed and evaluated by five project experts. Selection was based on the number of affected individuals in the family, the severity of their scoliosis curvatures, and the estimated genetic relationship between enrolled, affected individuals. Families with other musculoskeletal conditions or AIS from multiple sides of the family were excluded. Three to five affected individuals were selected for sequencing per family based on the degree of hypothesized genetic distance between them, availability of high-quality DNA, and severity of spinal curvature.

Pedigrees for these families and a summary of clinical information for all sequenced individuals are provided in Supplementary Files S1 and S2. On the pedigrees, degree of spinal curvature as measured by Cobb angle is indicated in numbers below affected individuals (i.e., 20D). Individuals with known double curves have both listed (i.e., 26/30D), as are any triple curvatures. All individuals with listed curvatures were examined radiographically, and any found to be unaffected on physical exam are labeled as “confirmed negative”. Proband for each family is indicated with an arrow. 

### 2.3. Whole Exome Sequencing

Exome capture was completed using 1 µg of genomic DNA from 86 individuals across 23 families using the Agilent SureSelect Human V5 (51 Mb) exon capture kit. Samples were sequenced with a 2 × 100 bp run on an Illumina HiSeq 2500 at the Otogenetics Corporation facility in Atlanta, GA, with a minimum average coverage of 50X guaranteed per sample.

### 2.4. Bioinformatic Filtering

Whole exome reads were aligned to GRCh38 and variants were identified with FreeBayes as previously described [38,46]. Candidate variants were filtered by SnpEff (version 4.1g) [50] along with custom scripts to retain only non-synonymous SNPs, coding indels, and variants affecting splice sites. Known artifacts and variants whose frequency was greater than 0.05 in the ExAC database (r0.3) [51] were also stripped. If the variant was annotated in the dbNSFP database (version 3.0) [52,53], it was retained only if at least one of the included variant prediction algorithms (SIFT, Polyphen2, LRT, MutationTaster) scored it as “damaging”, signifying that the resulting change to the encoded protein had a predicted functional consequence. Variants that were not shared by all sequenced members of the family were not retained. Variants with a Minor Allele Frequency (MAF) < 0.05 that remained after the above filters were applied were retained for further analysis in separate gene sets. This MAF threshold was intentionally set higher than typical rare variant thresholds to account for the prevalence of the disease (2–3% of the general population), working under the hypothesis that low frequency variants may contribute to the high prevalence of this disease.

### 2.5. Genotyping

Variants appearing in multiple families that were present in the GO functional categories for “cytoskeleton” or “extracellular matrix” (or related terms) were prioritized for genotyping. Additional enrolled affected and unaffected family members were sequenced at the variant site by the Sanger method to establish whether the variant segregated with the AIS phenotype.

PCR was conducted in 20 µL reactions containing 10 µL Premix D (Epicentre Biotechnologies, Madison, WI, USA), 0.2 µL Taq Polymerase (Sigma, St. Louis, MO, USA), 60 ng genomic DNA, and 10 µM Forward and Reverse Primers. PCR reactions were run on a SimpliAmp Thermocycler (Fisher Scientific, Waltham, MA, USA) with a touchdown PCR protocol [38]. Primer sequences were obtained from Integrated DNA Technologies and are provided in Supplementary Files S2. Sanger sequencing was performed by Quintara Biosciences and chromatograms were analyzed using the CodonCode Aligner v9.0 (CodonCode Corporation, Centerville, MA, USA, https://www.codoncode.com/index.htm (accessed on 16 June 2021)).

Pedigrees for each sequenced family are provided in Supplementary Files S1 and were created using PedigreeXP software (PC Pal, https://www.pedigreexp.com (accessed on 16 June 2021)). 

### 2.6. Gene Set Overrepresentation Analyses

Gene set overrepresentation analyses (GOA) were performed on MAF < 0.05 gene lists with duplicate genes removed (*n* = 1160) from the combined list from each family. Both DAVID and EnrichR websites were used, as described below.

DAVID: DAVID (Database for Annotation, Visualization and Integrated Discovery) v6.8 was used to identify the significant GO terms and clusters in each gene list (https://david.ncifcrf.gov, accessed on 20 April 2021) [54,55]. The input gene list of 1160 genes resulted in 1146 DAVID IDs. Functional annotation clustering was used on our dataset with default settings and the GOTERM_All and GOTERM_CC_Direct annotation categories. 

Enrichr: The same input gene lists as used for DAVID were used in Enrichr, a gene enrichment software developed by the Ma’ayan laboratory [56,57] (https://maayanlab.cloud/Enrichr/, accessed on 27 April 2021). Additionally, family-specific gene lists were separately inputted into EnrichR. We report results from the 2018 GO term Cellular Component and KEGG pathways. Volcano plots and charts were generated with Appyter (https://appyters.maayanlab.cloud/#/, accessed on 27 April 2021). 

## 3. Results

To identify rare and low frequency variants associated with familial AIS, we performed whole exome sequencing on 23 multigenerational IS families (3–5 individuals per family, 86 individuals in total). Pedigrees for all families are provided in Supplementary Files S1, and clinical information for sequenced individuals is provided in Supplementary Files S2. Whole exome sequencing was performed using DNA extracted from whole blood, as described in the Methods. Illumina HiSeq reads were mapped to the human reference genome (hg39), and a minimum of 50X average coverage was obtained for each sample.

**Figure 2:**
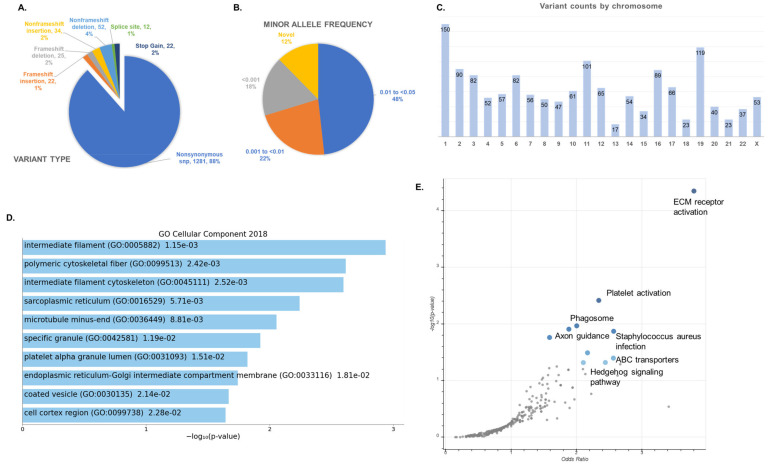
Summary information for total gene list (*n* = 1160) of familial AIS-associated variants across families with a minor allele frequency (MAF) <0.05 passing bioinformatic filtering, as described in the Methods. (**A**) Variant type with *n* variants and % of total provided. (**B**) Minor allele frequency of all variants using ExAC. (**C**) Variant counts by chromosome. (**D**) Top GO Cellular Component, 2018 terms using EnrichR. See Table 2 for top GO terms using DAVID. (**E**) Volcano plot of top enriched KEGG terms over expected.

We then filtered the list of variants, requiring that each retained variant was present in all sequenced members of the family, was predicted to be damaging, and had an ExAC minor allele frequency (MAF) of <0.05. This MAF filter thus included both low-frequency (MAF 1–5%) and rare variants (MAF < 1%). These filters resulted in a total of 1448 variants in 1160 genes across the 23 families, with 11 to 128 variants identified in each family (median = 51 variants). Figure 2 provides a summary of variant information identified across all families. Nonsynonymous single nucleotide polymorphisms (SNPs) constituted the majority of filtered variants (88%, *n* = 1281), followed by non-frameshift deletions (4%, *n* = 52), non-frameshift insertions (2%, *n* = 34), frameshift deletions (2%, *n* = 25), stop gains (2%, *n* = 22), frameshift insertions (2%, *n* = 22), and splice sites (1%, *n* = 12). About half (48%) of variants had a minor allele frequency (MAF) of 0.01–0.05, 22% had an MAF 0.001–0.01, 18% had an MAF < 0.001, and 12% were predicted to be novel. Chromosomes with the largest number of variants were chromosome 1 (*n* = 150) and chromosome 19 (*n* = 119). Full details of each variant, sorted by family, are provided in Supplementary Files S3.

We next searched for genes containing variants in multiple families, with the hypothesis that shared genes would be more likely to contribute to the IS phenotype. Most variant-containing genes (*n* = 1028 of 1160 total, 88.6%) were specific to only one family. 132 genes were shared by at least 2 families, 38 were shared between 3+ families, and 10 were shared by 4+ families. No genes were shared among all families. Table 1 provides a list of genes found within multiple families. Of this list, four genes had a previously observed association with either idiopathic, degenerative, or infantile scoliosis (*DLL3* [58,59], *AHNAK* [60], *TTN* [61], *ANKRD11* [62]).

**Table 1:** Genes containing variants in multiple idiopathic scoliosis (IS) families. Genes with the same variant identified across families are indicated with an *. Citations are given for genes previously associated with scoliosis (including idiopathic, degenerative, or infantile). Full variant results by family are provided in Supplementary Files S3.

*N* Families Containing Variant	Gene Names
2 families	*TMEM52, GCFC2, RTP5, SLC10A6, SAMD9L, PTPRD, ASCL1, GALC, CD34, ALPPL2, FAM189B, NTRK1, CHIT1, DCBLD2, COL6A5, ATG9B, NUTM2F, NME3, GFAP, KRTAP10-5, ZNF644, FLG, OR6P1, CSMD1, UQCRB, LMNTD2, THAP11, MLLT1, OR14A2, VWA3B, CFC1B, MSH3, AKAP3, GNB1L, RAB36, FGD1, THOC3, TNXB, AHNAK2, XYLT1, APOL3, CACFD1, RPL3L, PIGT, CTNNA3, LRIT1, TYMP, ASXL2, GPAT2, AARD, MGA, LGALS9C, C18orf65, AHCY, CSMD2, VPS41, BHLHE22, CCDC68, SYNJ1, HMGCS2, SYN2, DNASE1L3, CASP12, FSIP2, TET2, FAM153B, FAM153A, PFAS, PDZD2, KLHL32, ANKRD18A, MCM8, NPHP4, USP32, COL21A1, SSTR5, CEACAM21, FAM26F, OR4A5, KRT81, BDNF, SLC1A7, SPTA1, TNK2, PTPRE, CYB5R2, MTUS2, FANCA, KATNAL2, OTC, GPIHBP1, CLEC18B, SLC22A31, SETDB1*
3 families	*GPRIN1, SKIV2L, PRR23D1, TBC1D26, KIR3DL1, TTN* [61], *KRTAP4-3, DLL3* [58,59], *OBSCN, KCNQ5, CCDC168, PRR25, ANKRD11* [62], *HRC, GPR179, USP26, FCGBP, KCNN3, CACNA1H, ANKRD30B, KIAA1875, MRC1, ACOT4, KIAA0556, ERCC6L, DYSF, CEP170, AHNAK* [60]
4 families	*POM121, PKD1L2, TMPRSS13, KRT2, ZNF717, PDE4DIP*
≥5 families	*AL589743.1, TPRX1, FAM47A, WIPF3*

Although few genes were shared across families, we hypothesized that AIS families may share an enrichment of variants in specific functional categories. To identify categories of genes which were overrepresented with damaging variants within the AIS families, we performed gene set overrepresentation analysis (GOA) of our resulting filtered gene lists. We entered the MAF < 0.05 gene lists from individual families and combined data from all families into DAVID to identify overrepresented gene ontologies (GO Terms) (Table 2). The most overrepresented GO Cell Component categories across all families using DAVID were “microtubule” (*p =* 1.62 × 10^4^, 1.98 fold enrichment), “slit diaphragm” (*p =* 8.88 × 10^4^, 10.66 fold enrichment), and “intermediate filament” (*p =* 8.88 × 10^4^, 2.57 fold enrichment). Categories within extracellular matrix (ECM) GO terms, including “proteinaceous extracellular matrix” and “collagen trimer” also showed mild enrichments (Table 2) and the top enriched KEGG term was “ECM receptor activation” (Figure 2E). A full list of all GO functional categories is provided in Supplementary Files S2. We also used GOA to analyze an overrepresentation of cytogenic bands, as specific chromosomal regions have been linked with IS development by linkage analyses [63,64,65,66,67,68,69,70,71]. The most overrepresented band was 16p13.3 (*p* < 0.001), as described in Supplementary Files S2. This region was previously identified as significant in familial linkage analyses of a large AIS familial cohort. A neighboring region, distal chromosome 16p11.2 duplication, has been recently identified as a significant risk factor for severe AIS.

**Table 2:** Overrepresented Gene Ontology, Cellular Component terms using the combined Minor Allele Frequency (MAF) < 0.05 gene list from all families (input *n* = 1160). The genes under the terms “cytoplasm” and “plasma membrane” are limited to 50 for brevity. The full list of enriched GO Terms (all GO annotation sets) is provided in Supplementary Files S2.

Term	Count	%	*p* Value	Genes	Fold Enrichment
microtubule	36	3.14	1.62 × 10 ^4^	*INVS, DNAH1, DNAH7, TUBAL3, DCTN1, DNAH6, IQGAP1, CAMSAP2, GOLGA2, TEKT1, DVL1, KIF13B, TEKT2, CEP170, KIF21B, FSD1, KIF1A, PCNT, DYNC2H1, DNAH14, MTUS2, KCNAB2, SHROOM1, HAUS5, EML2, DLG1, SYNJ1, KATNAL2, INCENP, KIF26A, KIFC1, FEZ1, EHHADH, TTLL11, EIF3A, GAS8*	1.98
slit diaphragm	5	0.44	6.761 × 10 ^4^	*TRPC6, KIRREL2, NPHS1, MAGI2, IQGAP1*	10.66
intermediate filament	17	1.48	8.88 × 10^4^	*FLG, DSP, KRTAP13-4, KRT2, KRTAP26-1, KRTAP27-1, KRT79, KRT10, GFAP, KRT28, KRT37, PKP2, KRTAP6-1, SYNC, NES, KRT6A, PRPH*	2.57
Z disc	17	1.48	1.42 × 10^3^	*PPP1R12A, SYNPO2, AHNAK2, ATP2B4, SLC4A1, NEB, ANK3, ADRA1A, IGFN1, RYR3, MYPN, TTN, OBSCN, HRC, PDE4B, SYNC, CRYAB*	2.46
spindle	17	1.48	1.85 × 10^3^	*INVS, DIDO1, SPAG8, NUMA1, DCTN1, DCTN3, HEPACAM2, HAUS5, EML2, INCENP, KIFC1, NUP85, CLTCL1, ANKRD53, CEP170, E4F1, KBTBD8*	2.40
cytoplasm	347	30.28	3.04 × 10^3^	*RPL5, ANKLE1, MTRR, HDAC10, RGSL1, ABCA12, BACH2, WDR87, ENDOV, TBK1, CYP2D7, MPRIP, PPP4R2, C7ORF31, FAM65C, PLCE1, TRIM26, RTTN, KIF21B, ADGB, SDS, BSX, KRT2, MTUS2, AFAP1, SCRIB, LCE1E, MAPK8IP2, EML2, PPP1R3G, INPP4B, KATNAL2, TEP1, FEZ1, ZSCAN26, WDPCP, TTLL11, FLYWCH1, ALPK2, PFN1, RIN1, TRIB2, KPRP, SPTBN5, PARPBP, DHX8, PCDH15, C1ORF198, AGAP1, XPC	1.13
lateral plasma membrane	10	0.87	3.37 × 10^3^	*CEACAM1, DLG1, KCNB1, MTCL1, ABCC6, PTPRO, DVL1, ANK3, IQGAP1, NKD2*	3.22
cytoskeleton	36	3.14	3.73 × 10^3^	*TENM1, DRC7, MTCL1, EVPL, PPL, GPHN, FGD1, SYNE1, KIAA0556, CNN2, AKAP12, EPB41L5, SGCD, EPB41L2, TNKS1BP1, PLEK2, NPHP4, CTNNA3, TRIM67, DSP, VASP, FARP2, AFAP1, FRMD4A, KCNAB2, PTPN13, ARHGAP24, APBB1IP, UBXN11, FRMD7, TRIP10, FILIP1, NF2, RIN1, PFN1, TRIB2*	1.66
neuron projection	25	2.18	6.37 × 10^3^	*TENM1, GPI, TENM2, TENM3, AHCY, TENM4, PTPRO, IQGAP1, STON2, PARK2, DVL1, CADM1, DTNBP1, ATP2B4, ANK3, PTPN13, SSTR5, SYNJ1, FRMD7, RAPGEF2, FAS, ATP13A2, NF2, PFN1, CPEB2*	1.80
centrosome	38	3.32	1.08 × 10^2^	*ERCC6L, NUMA1, DCTN1, VPS4B, DCTN3, HEPACAM2, CDC14A, RPGR, TTC28, CAMSAP2, PPP4R2, TMEM67, C7ORF31, CHEK1, PDE4B, CEP170, NPHP4, RTTN, NEK3, CCDC141, PCNT, ZNF322, DCAF13, LRRCC1, PPP1R12A, CEP135, DYX1C1, CCDC116, IFT140, CEP131, KLHL21, MTUS2, PDE4DIP, HAUS5, ALS2, CROCC, FEZ1, ALMS1*	1.52
axon	23	2.01	1.11 × 10^2^	*NTRK1, EPHA5, CNTNAP2, TENM3, KCNB1, PTPRO, DAB2IP, DTNBP1, IQGAP1, IGSF9, ROBO1, SPTA1, ALCAM, ALS2, FEZ1, DVL1, KIF13B, CHRNA10, KIF21B, NEK3, LDLRAP1, CRYAB, VPS16*	1.77
spectrin	4	0.35	1.29 × 10^2^	*SPTA1, SPTBN5, EPB41L2, SPTBN2*	7.58
sarcoplasmic reticulum	7	0.61	1.30 × 10^2^	*ATP2A3, ITPR1, CLEC18B, MRVI1, ANK3, XDH, RYR3*	3.51
plasma membrane	272	23.73	1.48 × 10^2^	*RGSL1, SLC4A1, ABCA12, ABRA, SLC4A5, PIEZO1, PLCE1, GPR179, DYNC2H1, EPHA5, IL15RA, UNC5A, OR1J4, KCNK13, CACNA2D2, SCRIB, ANK3, SYTL5, PHKA2, HSPG2, SYTL3, KEL, FEZ1, OR6C6, KCNQ5, WDPCP, RIN1, EPHA1, OR5AK2, CFB, TAS2R42, SLC22A1, PCDH15, MGST2, C2CD4A, OR10T2, XPC, PCDH12, IQGAP1, MST1R, PPL, IGSF9, OR52L1, ART1, EPB41L5, NCSTN, EPB41L2, PLCG2, KCNN3, APOB	1.13
proteinaceous extracellular matrix	25	2.18	2.55 × 10^2^	*FBN2, TNXB, LAMC3, ADAMTS12, ADAMTS10, ADAMTS14, GPC1, SLIT2, MUC4, IMPG1, TECTB, AMBN, FN1, MMP10, MMP16, CILP, COL4A3, COL4A6, COL8A2, COL21A1, COL9A3, COL6A5, COL9A2, MATN3, MATN2*	1.59
adherens junction	8	0.70	2.56 × 10^2^	*EPHA5, EPB41L5, CEACAM1, TNK2, PKP2, FMN1, CTNNA3, NF2*	2.73
sarcolemma	11	0.96	2.65 × 10^2^	*DLG1, SGCD, AHNAK, KCNB1, AHNAK2, DYSF, DTNBP1, ANK3, SYNC, SLC8B1, RYR3*	2.21
presynapse	9	0.79	3.25 × 10^2^	*SYT3, SYNJ1, DVL1, SYT15, SCRIB, STON2, SYTL5, SYTL3, PARK2*	2.40
apical plasma membrane	26	2.27	3.54 × 10^2^	*MTCL1, PTPRO, SIPA1L3, SLC4A5, PARD6B, DSTYK, SLCO2B1, CD34, DUOX2, GPIHBP1, SPTBN2, SLC22A11, MUC17, SLC10A2, ABCC6, AKR1A1, FN1, NRG1, ABCA7, CEACAM1, CDHR2, RAPGEF2, CHRFAM7A, WDPCP, SLC26A4, KCNK1*	1.52
collagen trimer	11	0.96	4.25 × 10^2^	*MSR1, COL27A1, COL7A1, COL4A3, COL8A2, SAAL1, COL4A6, COL21A1, COL9A2, COL6A5, OTOL1*	2.04

Next, we performed GOA on each family individually, to determine whether the cytoskeletal functional categories seen in the combined gene list were driven by a single family or many families. Overall, a cytoskeletal GO term was detected among the significantly enriched terms in every family except Family H, J, and O (Supplementary Files S2). Every family possessed at least one variant in a cytoskeletal gene. 

We next selected variants of interest for genotyping to determine whether the variant segregated with the AIS phenotype within each relevant family. Specific variants were genotyped if they were present in functional categories of interest (cytoskeletal or extracellular matrix GO terms), and if additional affected and unaffected members of the family were enrolled in our study. Ten genes (*ANKRD11, COL21A1, COL6A5, FGD1, NPHP4, OBSCN, TNXB, CTNNA3, NTRK1,* and *PDE4DIP*) containing multiple variants across families were genotyped. Of these, variants within *TNXB, CTNNA3, NTRK1,* and *PDE4DIP* showed segregation of the variant with the IS phenotype in additional affected and unaffected family members (Supplementary Files S2). *TNXB* (tenascin XB) localizes to the major histocompatibility region on chromosome 6 and encodes an ECM glycoprotein and has been associated to classic Ehlers-Danlos syndrome (EDS), a connective tissue disorder with scoliosis as one characteristic of the phenotype [72]. The *TNXB* variants within our dataset (Family G: TNXB:NM_019105:exon24:c.C8192G:p.P2731R; Family S: TNXB:NM_019105:exon12:c.G4444A: p.V1482M) do not appear in the EDS variant database to date [73,74,75]. *CTNNA3* (Catenin α 3) encodes a protein within the vinculin family of cell-cell adhesion proteins, and has been linked with certain types of cardiomyopathy [76]. *NTRK1* (Neurotrophic Receptor Tyrosine Kinase 1) encodes a protein that binds neurotrophin peptide and participates within neuronal cell differentiation and specification of neuronal cell subtypes [77]. *PDE4DIP* encodes myomegalin, which anchors phosphodiesterase 4D to the Golgi/centrosome regions. An isoform of myomegalin was recently shown to form a complex with AKAP9 and CDK5RAP to link the pericentrosomal complex to the microtubule-nucleating complex [78]. The remaining variants showed no segregation or only partial segregation of the variant with the AIS phenotype.

## 4. Discussion

In this study, we report an enrichment of predicted damaging variants in cytoskeletal and extracellular matrix (ECM) functional categories within adolescent idiopathic scoliosis (AIS) families. Additionally, this cohort showed minimal overlap in specific genes across families. Our results support and add to the growing evidence that AIS is a highly polygenic disorder [7,8,28,79,80] in which multiple variants of variable effect size, potentially in combination with epigenetic and environmental phenomenon [81], contribute to the disease phenotype. 

The ECM is a dynamic molecular network of proteoglycans, glycoproteins, minerals, and related proteins that plays a critical role within musculoskeletal tissues [82]. The ECM provides critical structural networks and scaffolding to tissues, and contributes to cellular signaling, growth, and repair [83]. Studies of genes encoding the “matrisome”, the proteins composing and relating to the ECM, have identified dozens of causal ECM mutations for connective tissue and musculoskeletal disorders [83]. A polygenic burden of rare variants in musculoskeletal collagen genes was linked to AIS risk in a large case-control cohort [28], and our group previously showed mild variant enrichments in our pilot exome sequencing study of five AIS families [46]. Candidate ECM genes including *HSPG2* [29] and *FBN2* [30] have also been linked to AIS in specific cohorts. Several collagen genes (*COL8A2, COL4A3, COL6A5, COL27A1, COL7A1, COL21A1, COL9A2, COL9A3, COL4A6*) and perlecan (*HSPG2*) appeared in our families (Supplementary Files S3). Candidate studies of ECM genes in AIS [63,84,85,86] were launched, in part, because scoliosis is a common phenotype of monogenic connective tissue disorders including Marfan and Ehlers-Danlos syndromes (EDS) [72,74]. *TNXB* (tenascin XB) variants, which have been associated with classic EDS, were found in two families in our current cohort as well as one family in our previous study [46]. The *TNXB* variant within Family G (TNXB:NM_019105:exon24:c.C8192G:p.P2731R) appeared in the Leiden Open Variation Database for EDS but was predicted as “benign” (https://databases.lovd.nl/shared/variants/0000313458#00021614, accessed on 4 May 2021). Functional data will be required to determine the pathogenicity of these variants. In a recent review, Wise et al. proposed that genetic variants affecting the cartilage matrisome, specifically of the intravertebral disc, may be involved in a subset of AIS cases [33]. Our results provide support to polygenic variants in ECM genes as a contributing factor to AIS in some family lineages.

The cytoskeleton comprises actin, microtubule, and intermediate filament networks and is responsible for a multitude of functions including molecular transport, cellular stability, molecular signaling, cell migration, and cell division [87]. The cytoskeleton and ECM are intricately connected within musculoskeletal tissues and play important roles in mechanotransduction, tissue stability and response to biomechanical loading [88,89]. Every family within our dataset had at least one cytoskeletal genetic variant, and 20/23 families showed an enriched cytoskeletal GO term (Supplementary Files S2). Variants in the centriolar protein *POC5* have been associated with scoliosis through human and animal studies [90,91,92]. The cytoskeletal kinesin *kif6* was shown to be necessary for proper spine development in zebrafish [34], and mutations in *kif6* also appeared in a zebrafish ENU genetic screen for scoliosis [93].

Defects in cilia, microtubule-based projections critical for cell signaling and fluid flow, have been linked to scoliosis in animal models [31,34,35,37,94,95]. A variant in the ciliary kinesin *KIF7* was found within one AIS family and specific *KIF7* mutations produced scoliosis in zebrafish [38]. Additionally, an overrepresentation of cilia variants was observed in our pilot exome sequencing cohort [46]. However, functional roles of the cytoskeleton and ECM in the development of IS have not yet been demonstrated in humans.

This study contains several limitations. Our familial cohort is relatively small and our ability to understand the impact of rare or low frequency variants in relation to the expression of a complex genetic disease, such as AIS, is limited [96]. Low frequency variants frequently lie outside the scope of large statistical association studies, and therefore may contribute to the “missing heritability” that accompanies many complex traits [97]. The majority of our discovered variants are heterozygous, and we do not know without functional testing whether these variants have a true dominant-negative effect on the resulting protein. To confirm our observed functional pathway enrichments, validate the presence of specific genetic variants, and obtain adequate statistical power, a larger cohort of AIS families must be sequenced. Additional functional studies of specific variants, with particular focus on those most likely to be damaging (i.e., stopgain, frameshift mutations) will be required to demonstrate causality [93].

Collectively, our results provide support to the hypothesis of ECM and cytoskeletal involvement in AIS etiology through what is, to our knowledge, the largest sequencing study of AIS families to date. These results suggest that there are many specific genes that can collectively increase disease risk, although there may be affected pathways that are shared across families. We hypothesize that (1) individual AIS families harbor low frequency mutations in different functional categories, resulting in different subsets of AIS, and/or (2) individuals with AIS require mutations in multiple gene categories (i.e., the cytoskeleton and ECM), resulting in mild dysfunction across several molecular pathways, to cause disease. Specifically, these results suggest that mild mutations in cytoskeletal or ECM genes may play a role in AIS etiology. Ultimately, this work will assist in the ability to predict the onset of adolescent idiopathic scoliosis and the risk of progressive disease and, thus, lead to the development of more personalized treatments for individuals with AIS. 

## 5. Conclusions

This work presents a novel set of candidate variants found in affected individuals from 23 AIS families. In agreement with our previous WES study of five AIS families, we observed an overrepresentation of variants in cytoskeletal and ECM functional categories, with few specific genes shared across families. Overall, this work paints a picture of AIS genetic etiology as highly polygenic and specific to individual family lineages. An analytical approach that integrates data from family-based sequencing with genetic association studies, an understanding of study population variation, population stratification and genetic heterogeneity, and advances in clinical phenotyping will enhance our ability to define the genetic complexity of this disorder. 

## Data Availability

The authors affirm that all data necessary for confirming the conclusions of this article are represented fully within the article and its supplementary files, including the complete lists of filtered variants for each family.

## Supplementary Materials

The following are available online at https://www.mdpi.com/article/10.3390/genes12060922/s1: File S1: Family Pedigrees. Degree of spinal curvature is indicated with a D (degrees). Individuals marked “confirmed positive” were confirmed as affected with AIS but were unable to provide us with an X-ray for measurement. Exome sequenced individuals are indicated. File S2: Table S1: Clinical information from all exome sequenced individuals; Table S2: Enriched cytobands from MAF < 0.05 gene list; Table S3: Genotyping of genes in multiple families and in functional categories of interest; Table S4: Full Gene Ontology (GO) Term enrichment results (*p* < 0.01) for all functional categories using DAVID; File S3: Detail of all filtered variants, sorted by family. All filtered variants had a minor allele frequency (MAF) < 0.05, were present in all sequenced members of the family, and were predicted to be damaging to the resulting protein by at least one algorithm. Details of the exome sequencing strategy and bioinformatic filtering process are provided in the Methods.
