# Supplementary material for: Whole Exome Sequencing of 23 Multigeneration Idiopathic Scoliosis Families Reveals Enrichments in Cytoskeletal Variants, Suggests Highly Polygenic Disease
Source: Genes (Basel). 2021 Jun 16;12(6):922. doi: 10.3390/genes12060922 (PMC8235452; doi:10.3390/genes12060922)
Supplement: Supplementary file 1 [file genes-12-00922-s001.zip › genes-1246102-supplementary-final/File S1 v2.pdf]

Supplemental File 1: Pedigrees for all exome sequenced families with AIS. Individuals affected with AIS are indicated with filled shapes, and individuals selected for sequencing are indicated by "exome-seq" below affected individuals. Degree of spinal curvature as measured by Cobb angle is indicated in numbers below affected individuals (i.e. 20D). Individuals with known double curves have both listed (i.e. 26/30D), as are any triple curvatures. All individuals with listed curvatures were examined radiographically, and any found to be unaffected on physical exam are labeled as "confirmed negative". Proband for each family is indicated with an arrow.

Family A

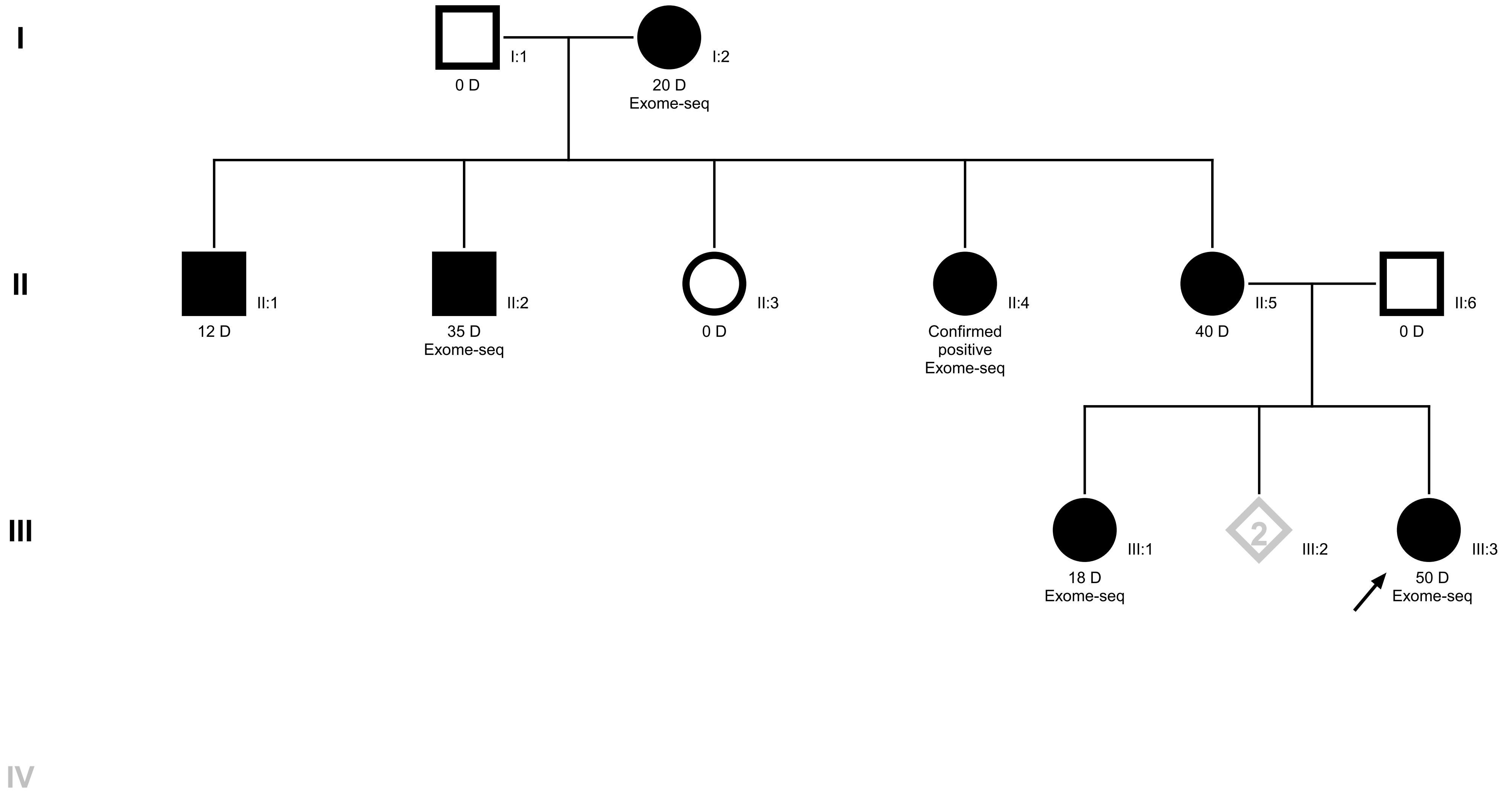

I
II
III
IV
I:1
0 D
I:2
20 D
Exome-seq
II:1
12 D
II:2
35 D
Exome-seq
II:3
0 D
II:4
Confirmed positive Exome-seq
II:5
40 D
II:6
0 D
III:1
18 D
Exome-seq
2
III:2
III:3
50 D
Exome-seq

Family B

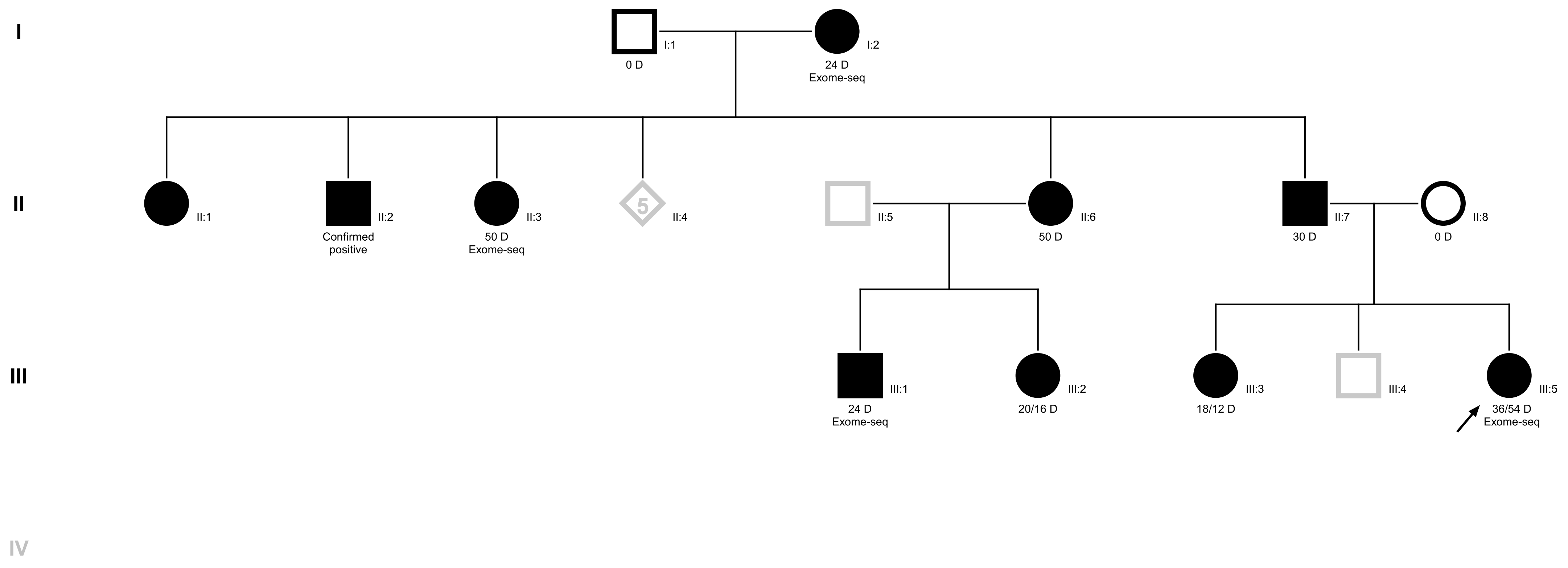

PC PAL
I
II
III
IV
I:1
0 D
I:2
24 D
Exome-seq
II:1
II:2
Confirmed
positive
II:3
50 D
Exome-seq
5
II:4
II:5
II:6
50 D
II:7
30 D
II:8
0 D
III:1
24 D
Exome-seq
III:2
20/16 D
III:3
18/12 D
III:4
III:5
36/54 D
Exome-seq

Family C

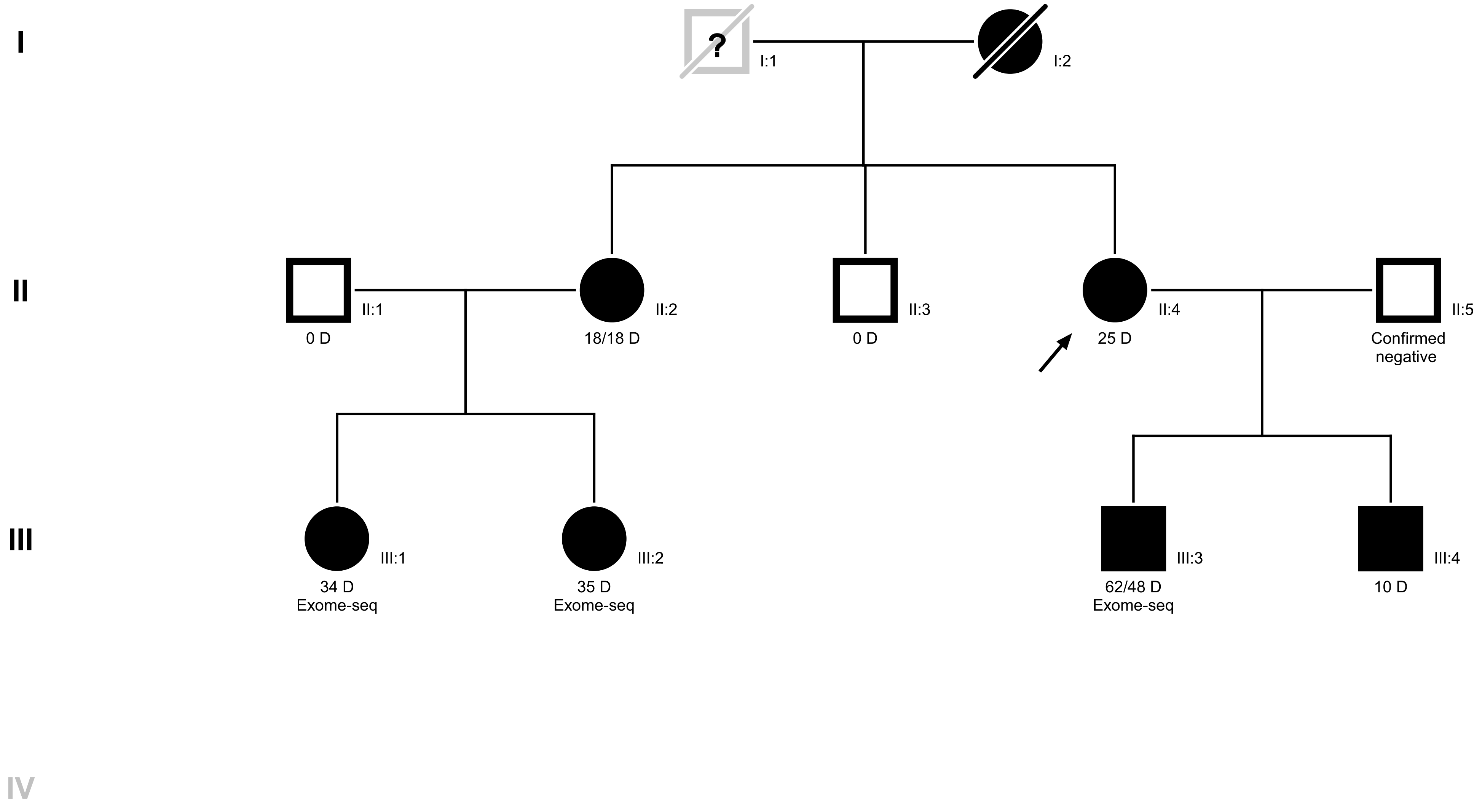

I
II
III
IV
?
I:1
I:2
II:1
0 D
II:2
18/18 D
II:3
0 D
II:4
25 D
II:5
Confirmed negative
III:1
34 D
Exome-seq
III:2
35 D
Exome-seq
III:3
62/48 D
Exome-seq
III:4
10 D

Family D

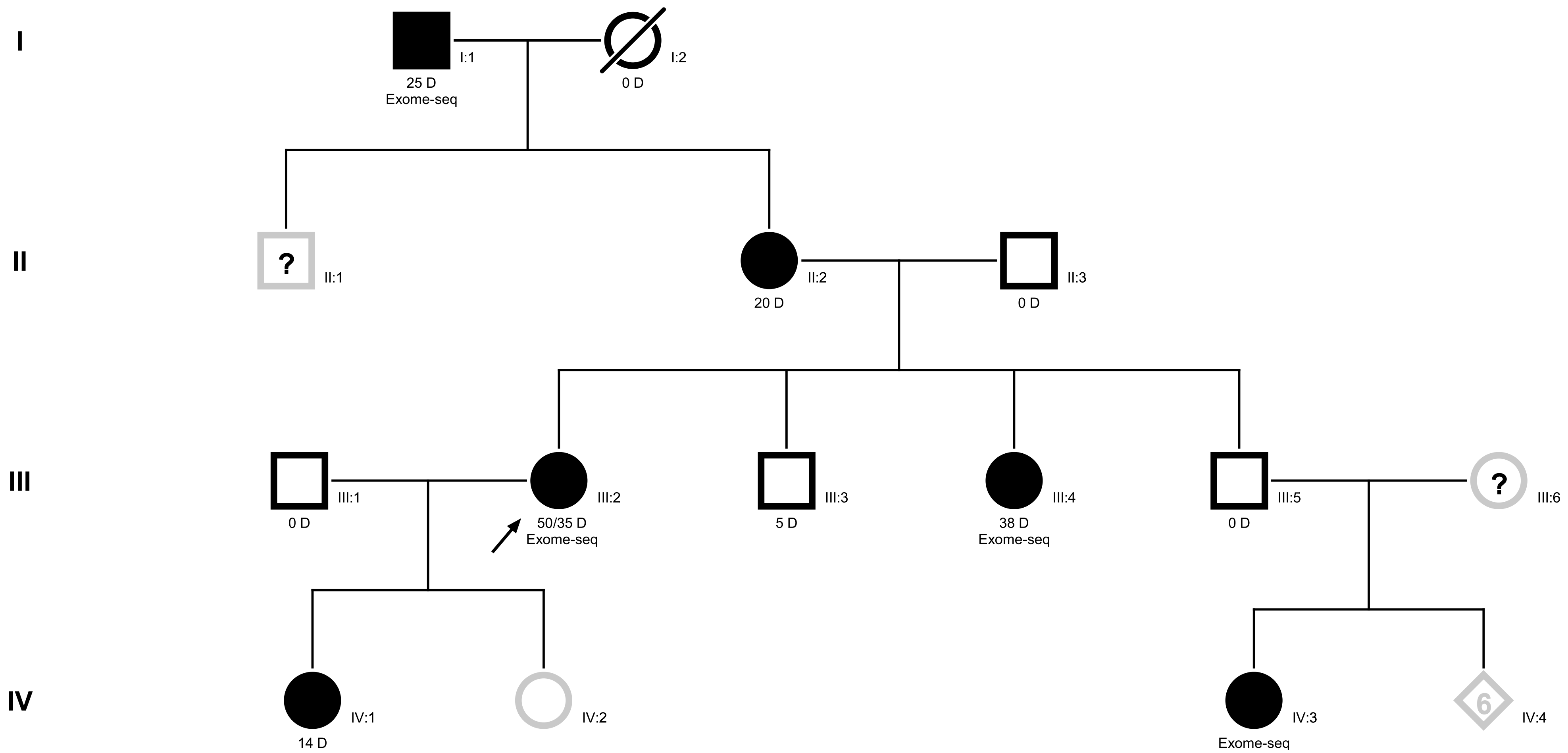

I
II
III
IV
I:1
25 D
Exome-seq
I:2
0 D
?
II:1
II:2
20 D
II:3
0 D
III:1
0 D
III:2
50/35 D
Exome-seq
III:3
5 D
III:4
38 D
Exome-seq
III:5
0 D
?
III:6
IV:1
14 D
IV:2
IV:3
Exome-seq
6
IV:4

Family E

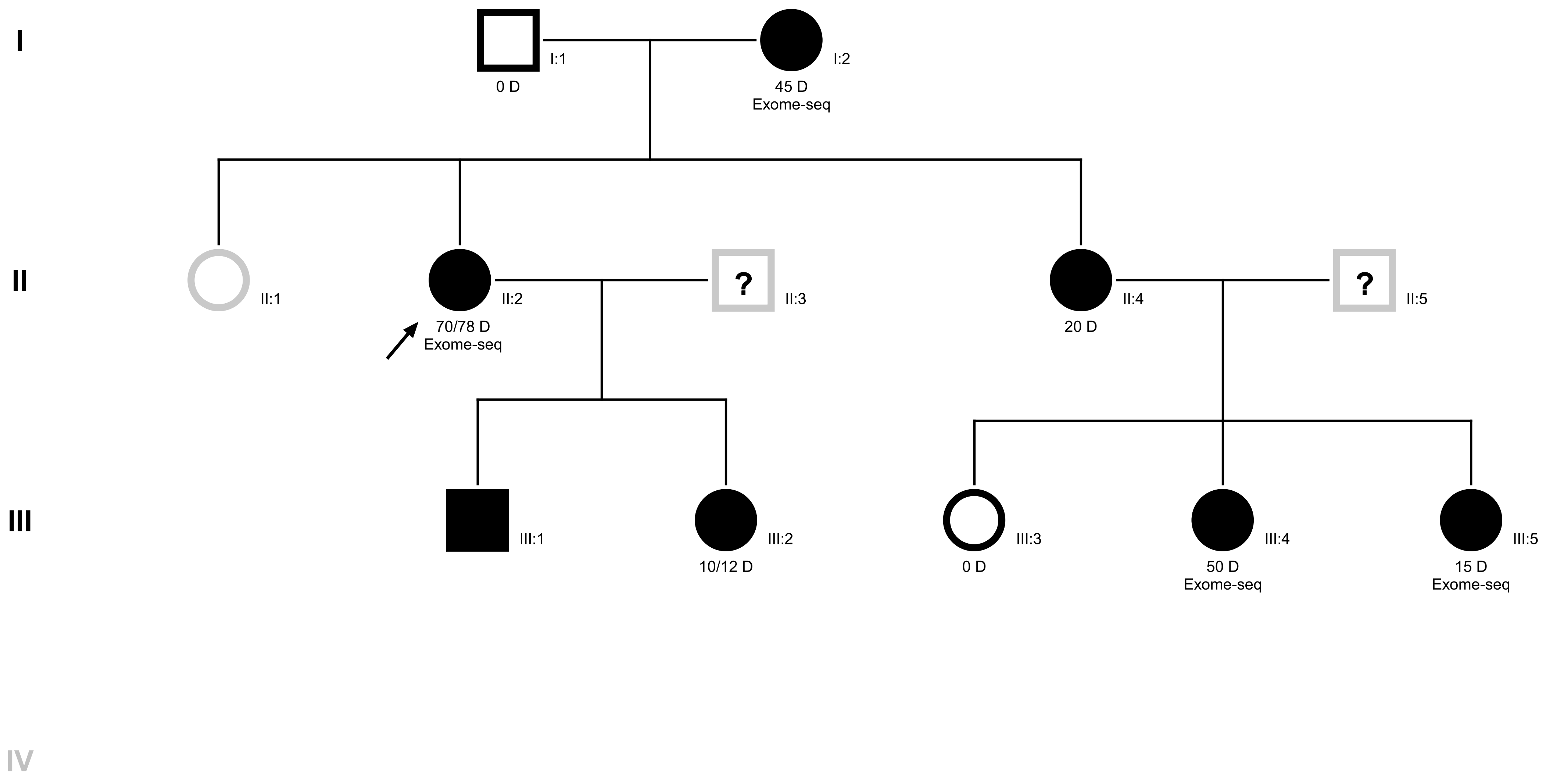

I
II
III
IV
I:1
0 D
I:2
45 D
Exome-seq
II:1
II:2
70/78 D
Exome-seq
?
II:3
II:4
20 D
?
II:5
III:1
III:2
10/12 D
III:3
0 D
III:4
50 D
Exome-seq
III:5
15 D
Exome-seq

Family F

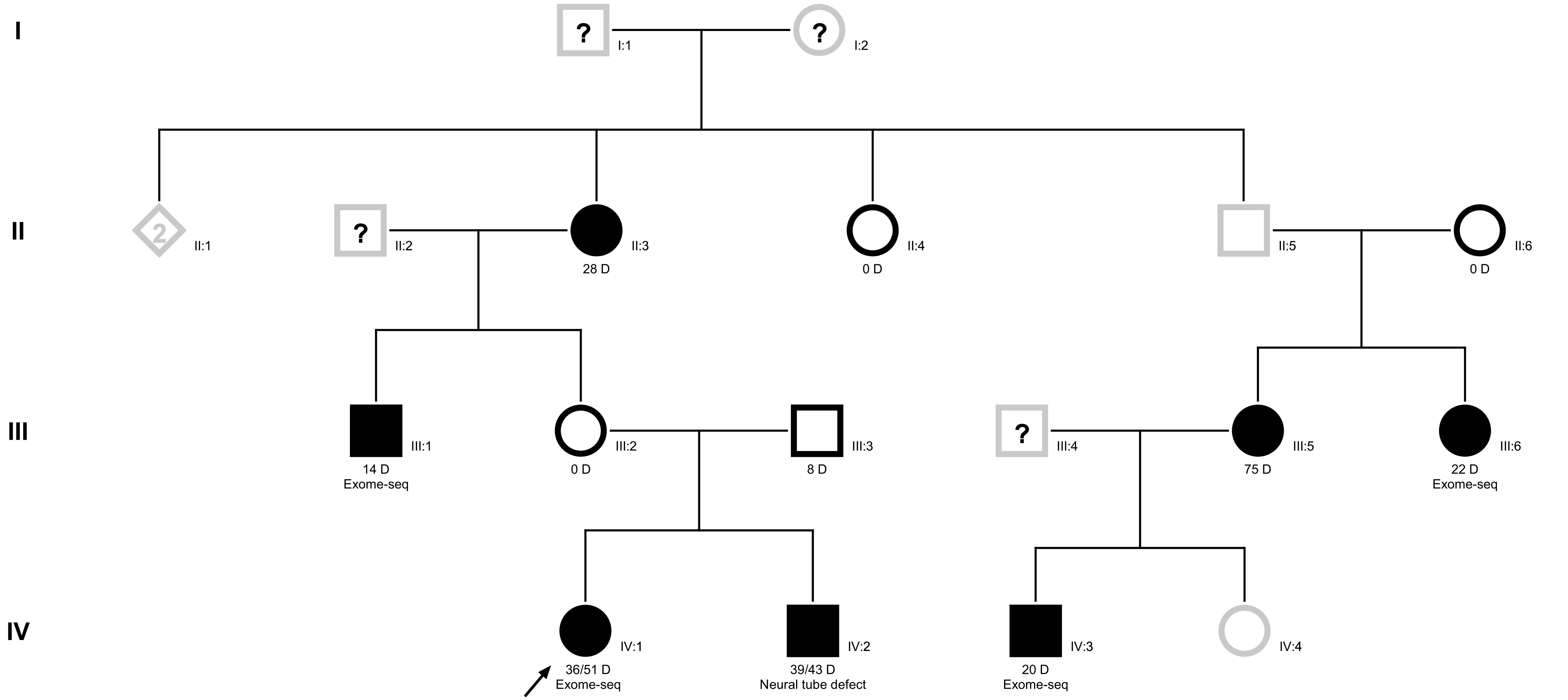

PC PAL
I
II
III
IV
?
I:1
?
I:2
2
II:1
?
II:2
II:3
28 D
II:4
0 D
II:5
II:6
0 D
III:1
14 D
Exome-seq
III:2
0 D
III:3
8 D
?
III:4
III:5
75 D
III:6
22 D
Exome-seq
IV:1
36/51 D
Exome-seq
IV:2
39/43 D
Neural tube defect
IV:3
20 D
Exome-seq
IV:4

Family G

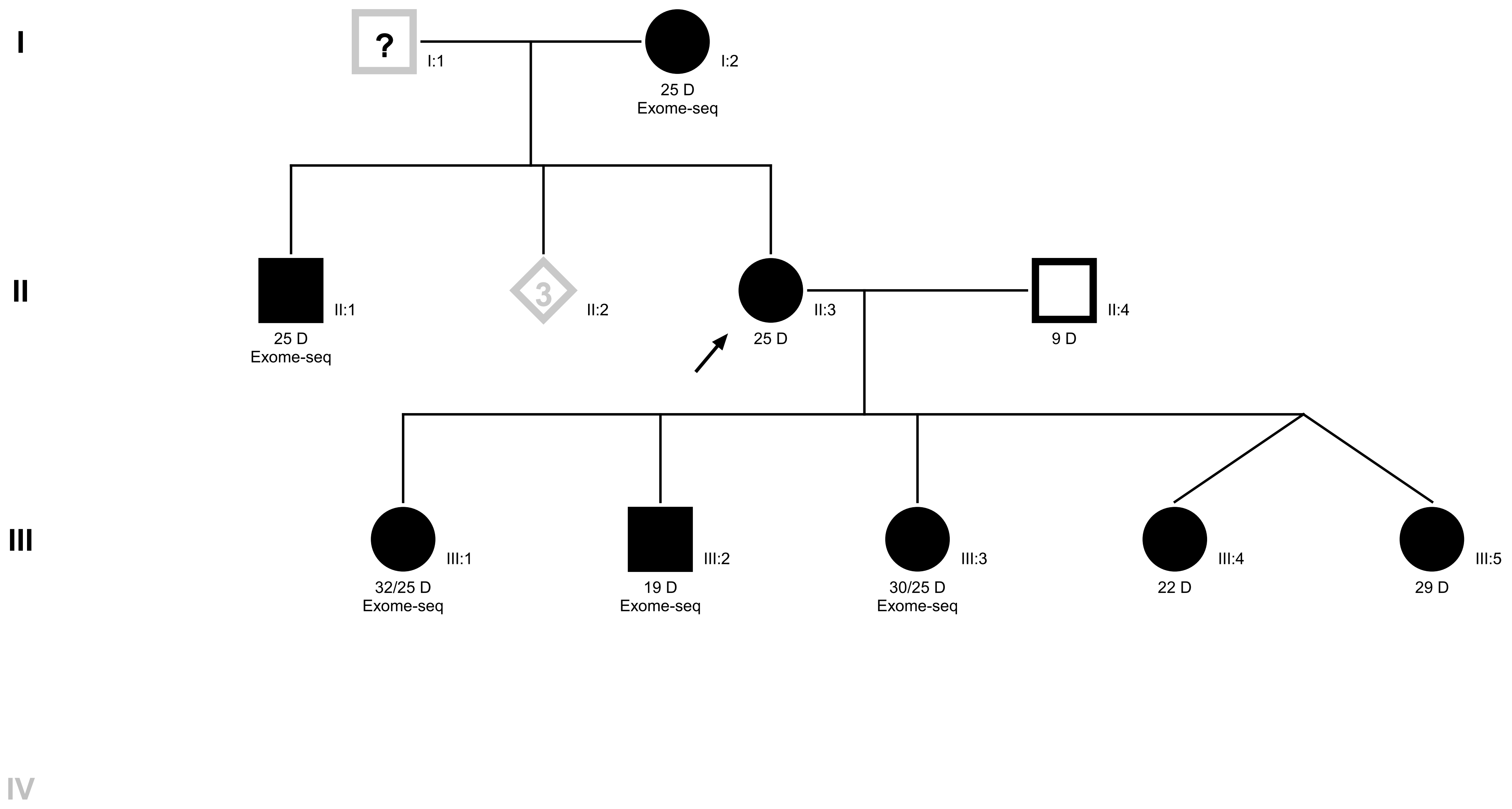

I
II
III
IV
?
I:1
I:2
25 D
Exome-seq
II:1
25 D
Exome-seq
3
II:2
II:3
25 D
II:4
9 D
III:1
32/25 D
Exome-seq
III:2
19 D
Exome-seq
III:3
30/25 D
Exome-seq
III:4
22 D
III:5
29 D

Family H

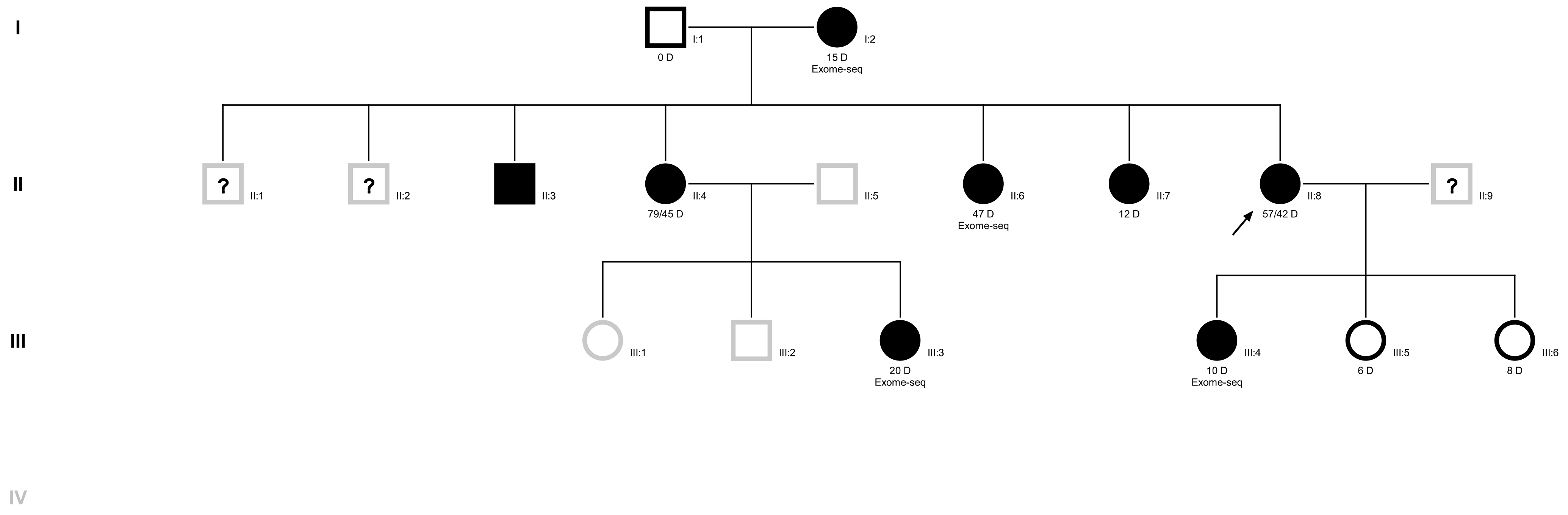

PC PAL
I
II
III
IV
I:1
0 D
I:2
15 D
Exome-seq
II:1
II:2
II:3
II:4
79/45 D
II:5
II:6
47 D
Exome-seq
II:7
12 D
II:8
57/42 D
II:9
III:1
III:2
III:3
20 D
Exome-seq
III:4
10 D
Exome-seq
III:5
6 D
III:6
8 D

Family I

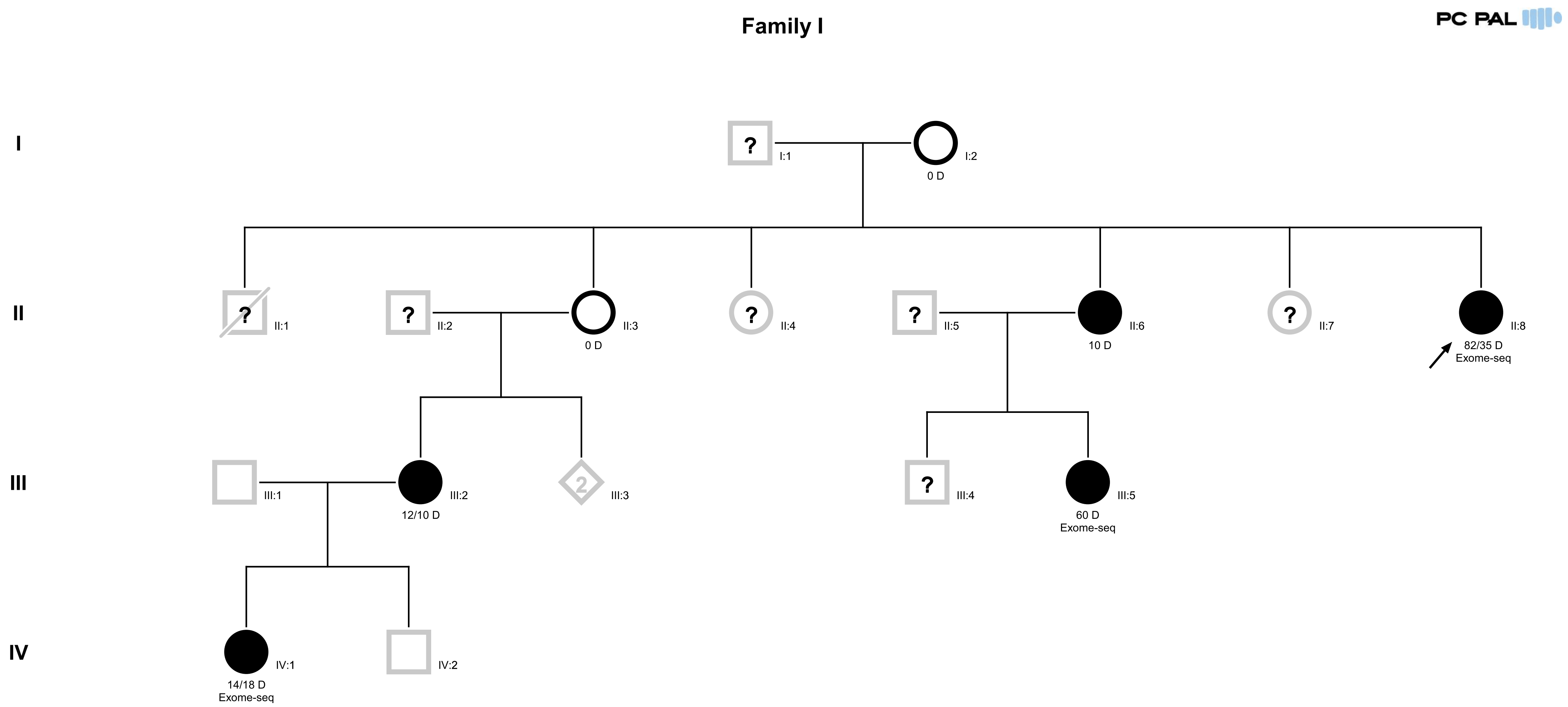

PC PAL
I
II
III
IV
?
I:1
I:2
0 D
II:1
II:2
II:3
0 D
II:4
II:5
II:6
10 D
II:7
II:8
82/35 D
Exome-seq
III:1
III:2
12/10 D
2
III:3
III:4
III:5
60 D
Exome-seq
IV:1
14/18 D
Exome-seq
IV:2

Family J

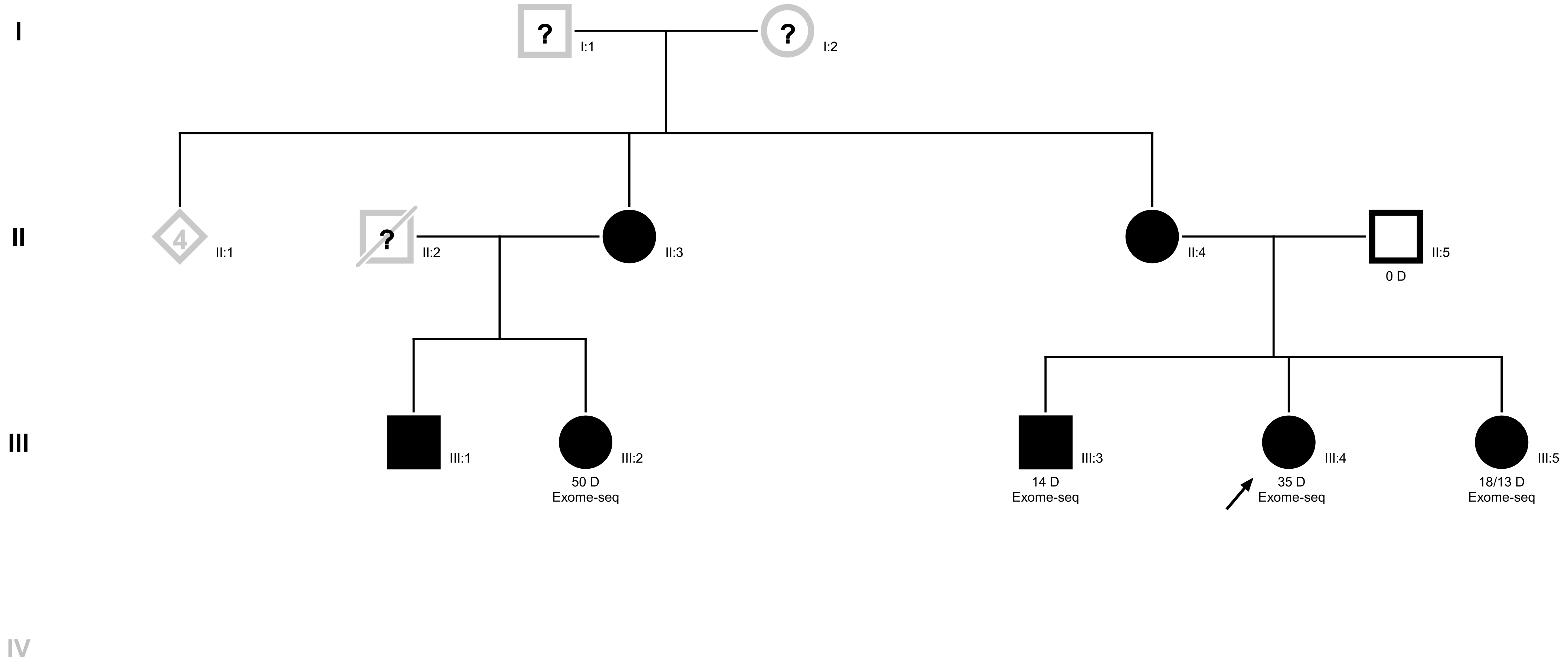

I
II
III
IV
?
I:1
?
I:2
4
II:1
?
II:2
II:3
II:4
II:5
0 D
III:1
III:2
50 D
Exome-seq
III:3
14 D
Exome-seq
III:4
35 D
Exome-seq
III:5
18/13 D
Exome-seq

Family K

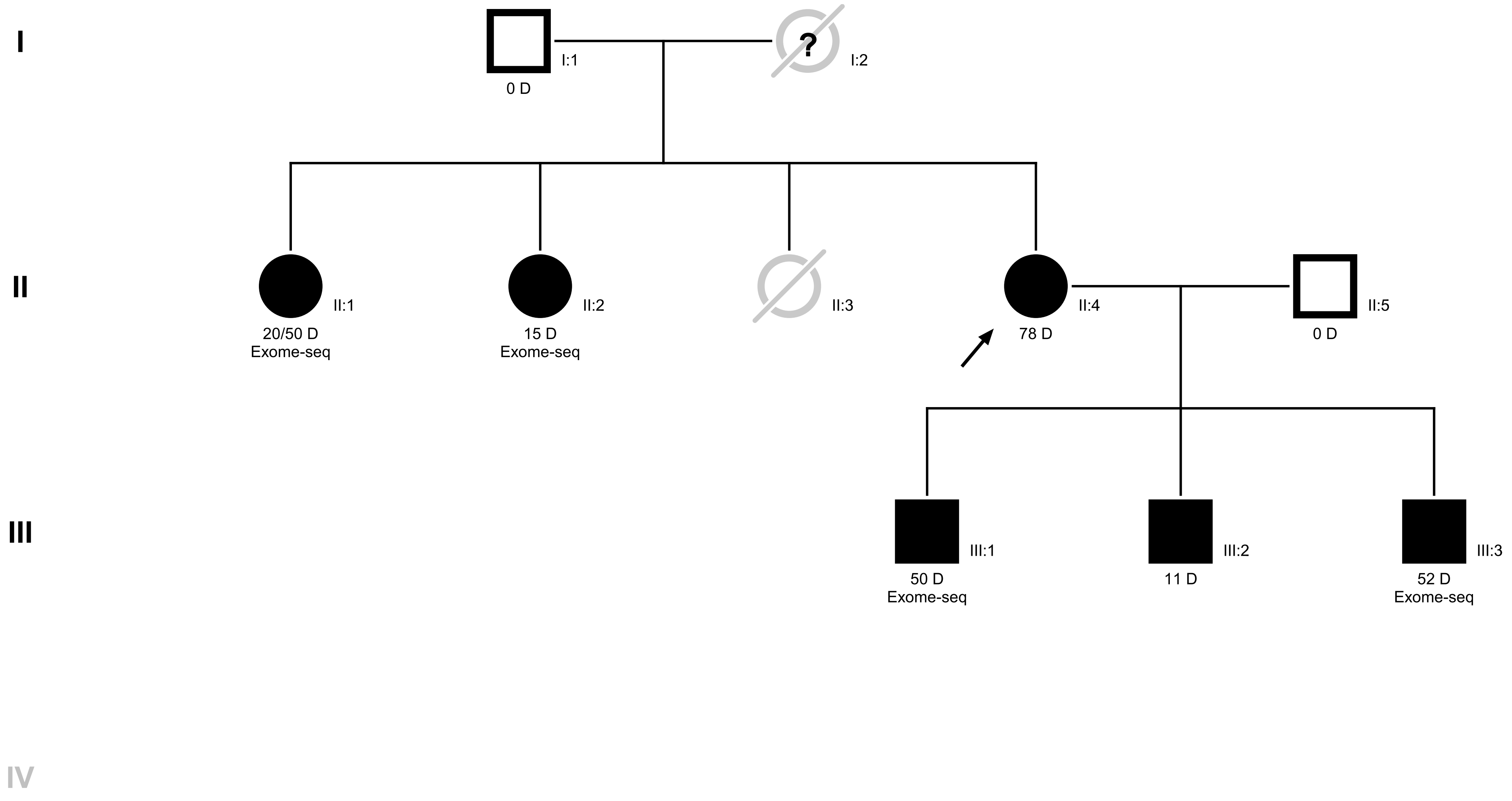

I
II
III
IV
I:1
0 D
?
I:2
II:1
20/50 D
Exome-seq
II:2
15 D
Exome-seq
II:3
II:4
78 D
II:5
0 D
III:1
50 D
Exome-seq
III:2
11 D
III:3
52 D
Exome-seq

Family L

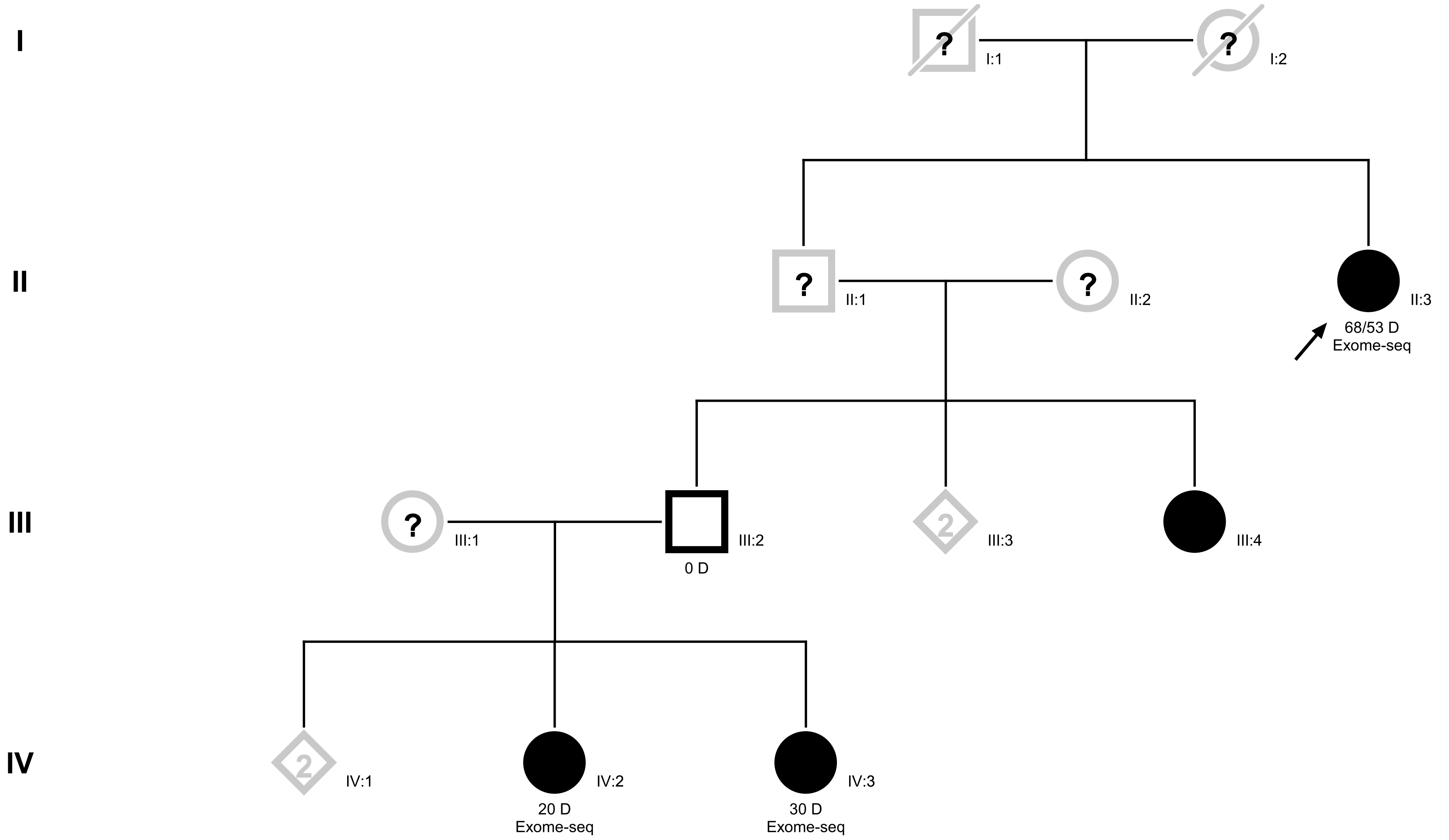

I
II
III
IV
?
I:1
?
I:2
?
II:1
?
II:2
II:3
68/53 D
Exome-seq
?
III:1
III:2
0 D
2
III:3
III:4
2
IV:1
IV:2
20 D
Exome-seq
IV:3
30 D
Exome-seq

Family M

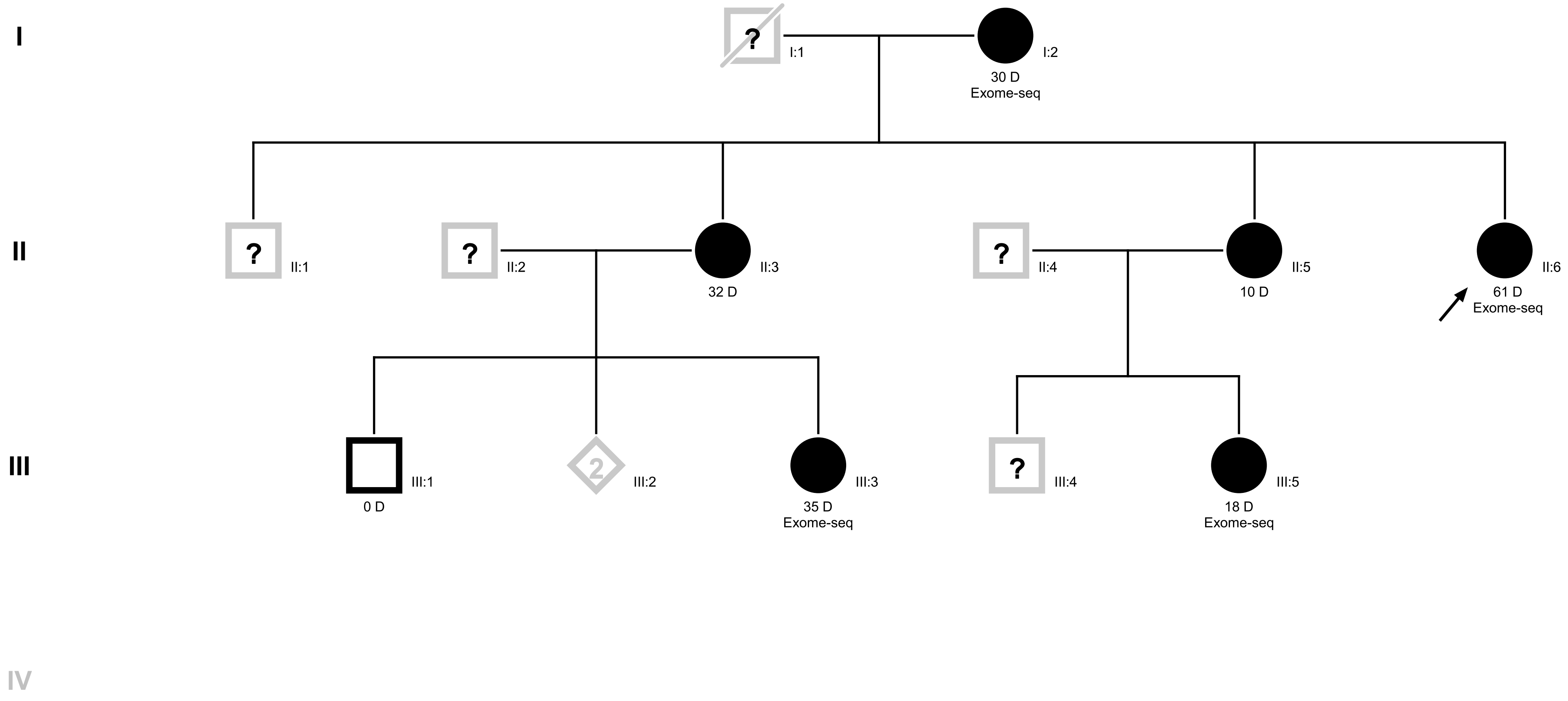

I
II
III
IV
?
I:1
I:2
30 D
Exome-seq
?
II:1
?
II:2
II:3
32 D
?
II:4
II:5
10 D
II:6
61 D
Exome-seq
III:1
0 D
2
III:2
III:3
35 D
Exome-seq
?
III:4
III:5
18 D
Exome-seq

Family N

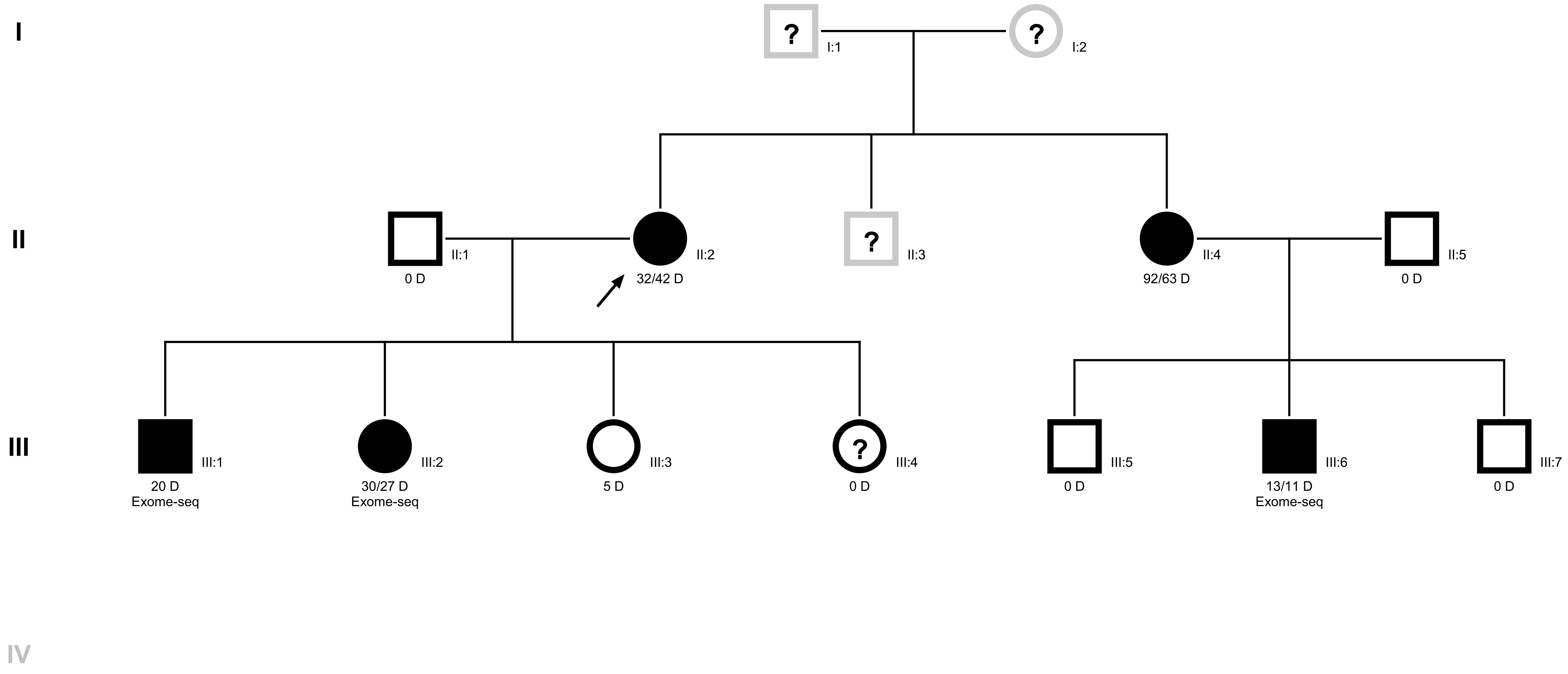

I
II
III
IV
?
I:1
?
I:2
II:1
0 D
II:2
32/42 D
?
II:3
II:4
92/63 D
II:5
0 D
III:1
20 D
Exome-seq
III:2
30/27 D
Exome-seq
III:3
5 D
?
III:4
0 D
III:5
0 D
III:6
13/11 D
Exome-seq
III:7
0 D

Family O

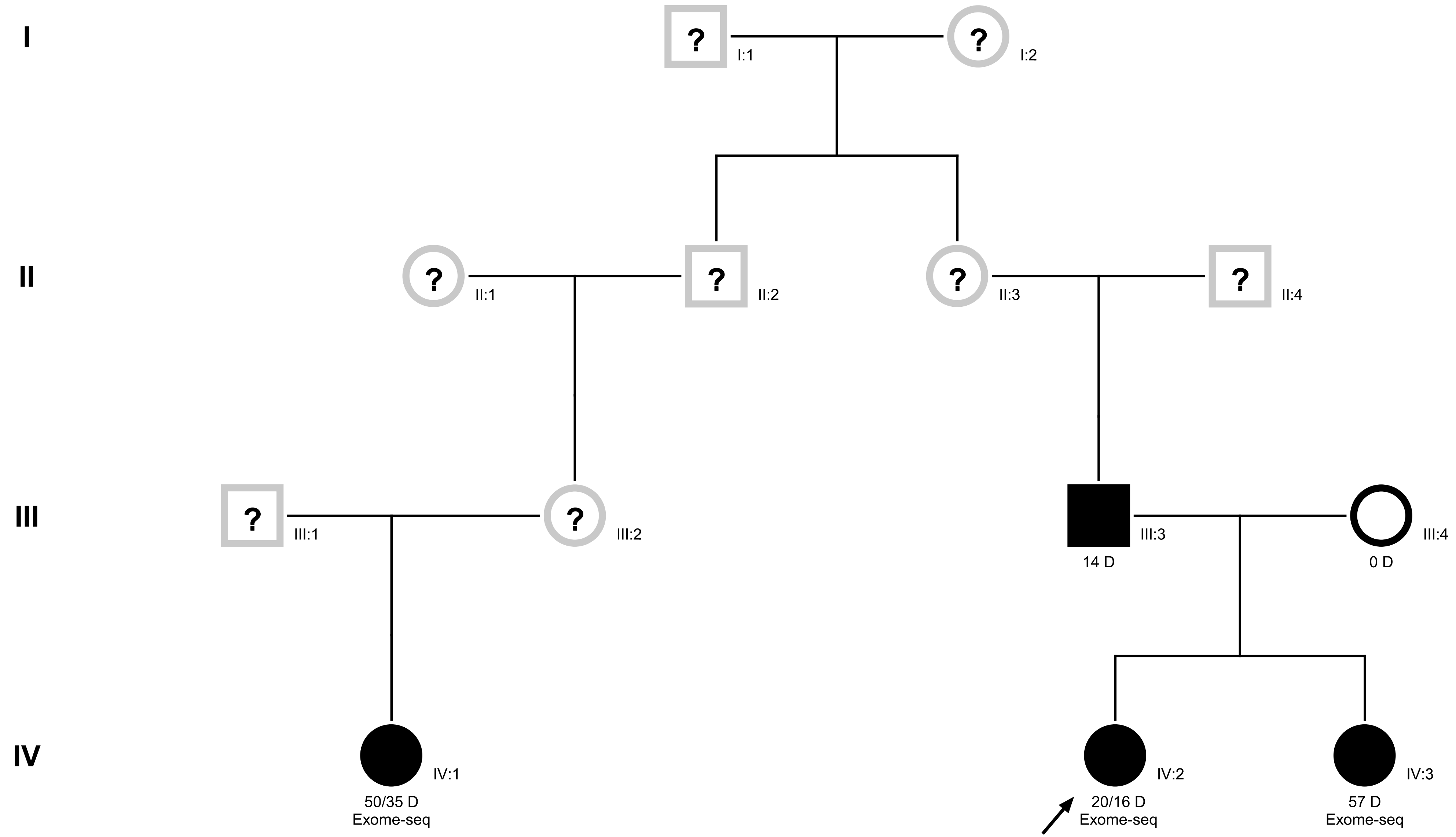
I
II
III
IV
?
I:1
?
I:2
?
II:1
?
II:2
?
II:3
?
II:4
?
III:1
?
III:2
III:3
14 D
III:4
0 D
IV:1
50/35 D
Exome-seq
IV:2
20/16 D
Exome-seq
IV:3
57 D
Exome-seq

Family P

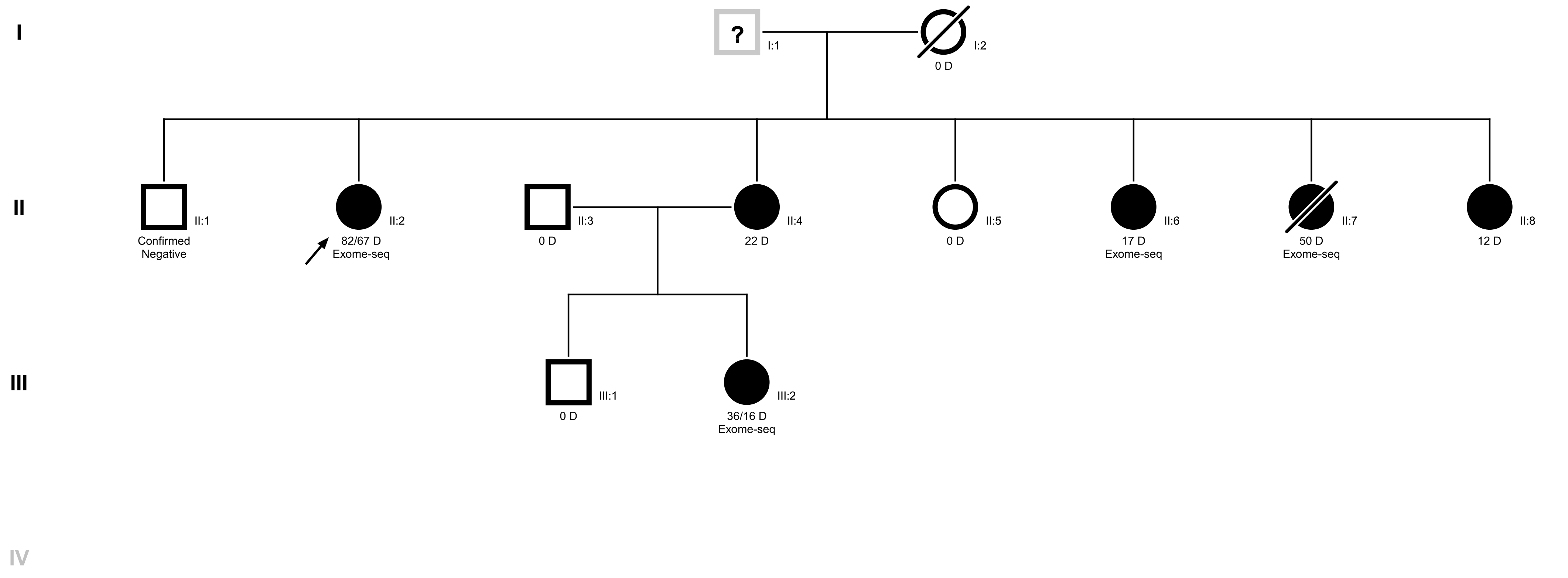

I
II
III
IV
?
I:1
I:2
0 D
II:1
Confirmed
Negative
II:2
82/67 D
Exome-seq
II:3
0 D
II:4
22 D
II:5
0 D
II:6
17 D
Exome-seq
II:7
50 D
Exome-seq
II:8
12 D
III:1
0 D
III:2
36/16 D
Exome-seq

Family Q

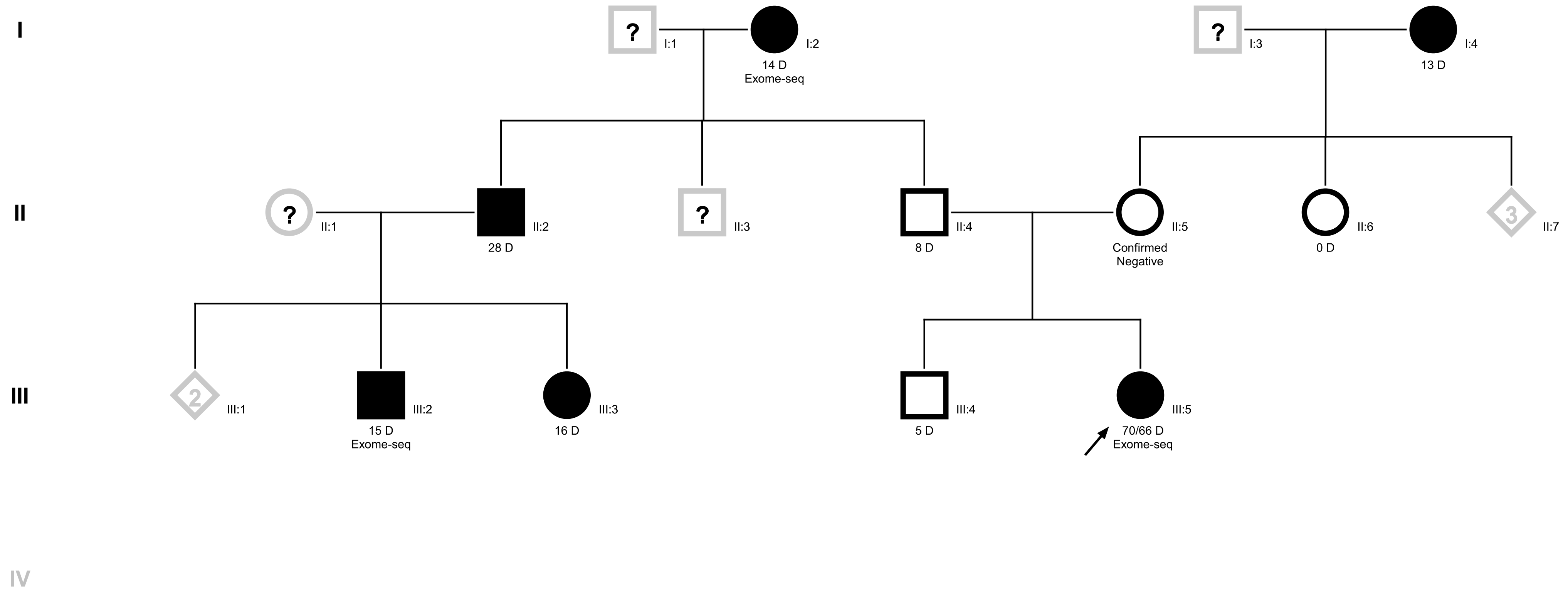

I
II
III
IV
?
I:1
I:2
14 D
Exome-seq
?
I:3
I:4
13 D
?
II:1
II:2
28 D
?
II:3
II:4
8 D
II:5
Confirmed
Negative
II:6
0 D
3
II:7
2
III:1
III:2
15 D
Exome-seq
III:3
16 D
III:4
5 D
III:5
70/66 D
Exome-seq

Family R

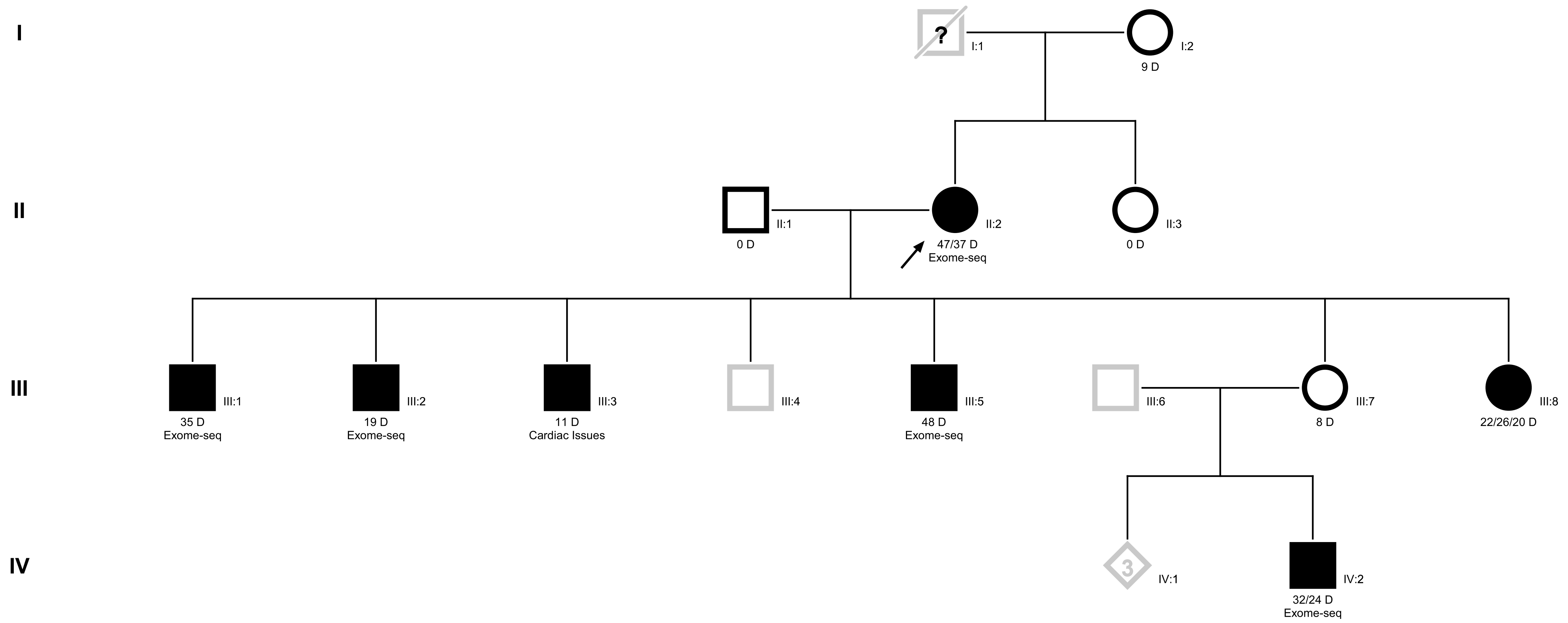

I
II
III
IV
?
I:1
I:2
9 D
II:1
0 D
II:2
47/37 D
Exome-seq
II:3
0 D
III:1
35 D
Exome-seq
III:2
19 D
Exome-seq
III:3
11 D
Cardiac Issues
III:4
III:5
48 D
Exome-seq
III:6
III:7
8 D
III:8
22/26/20 D
3
IV:1
IV:2
32/24 D
Exome-seq

Family S

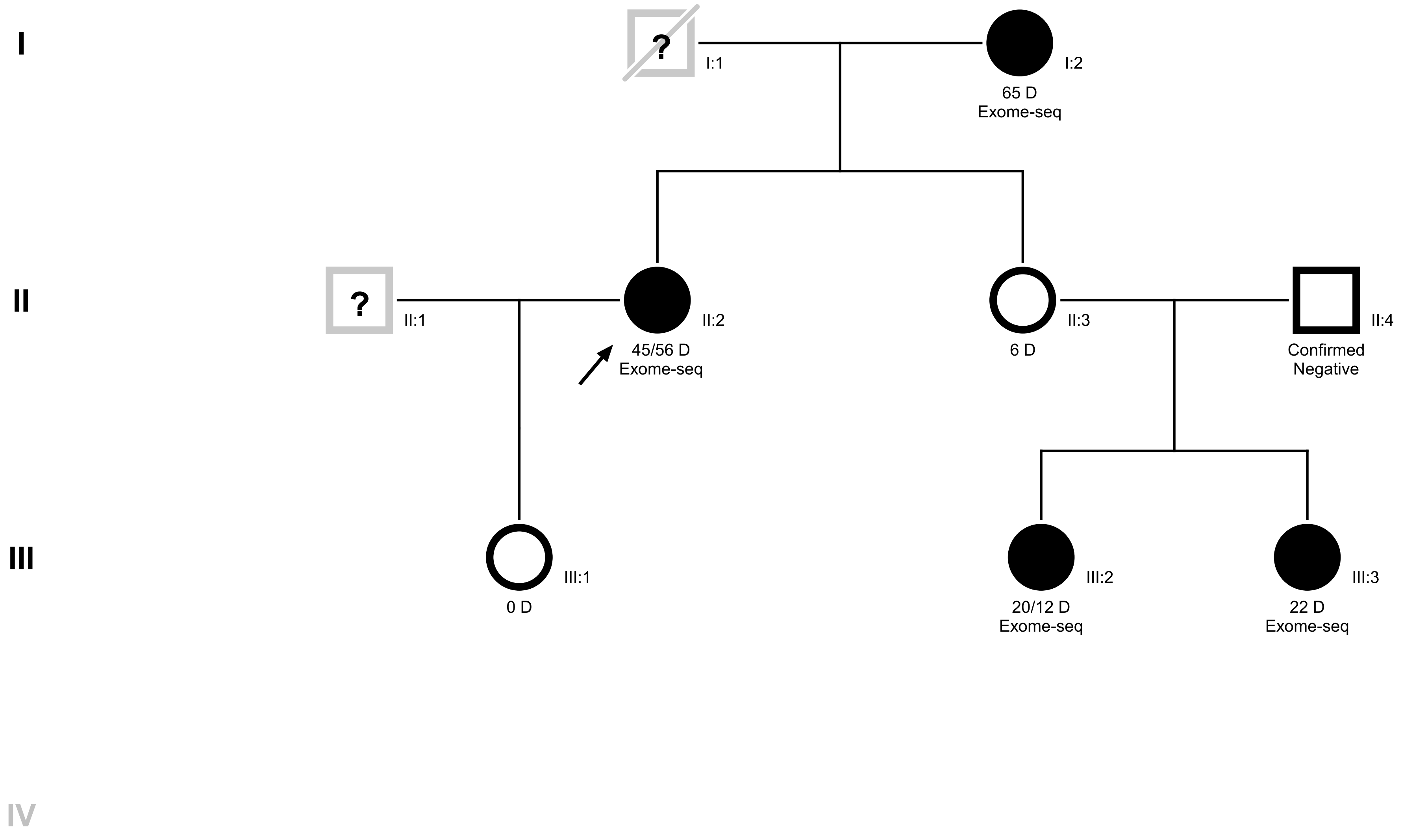

PedigreeXP 2.5.0.196 © PC PAL 2021

Family T

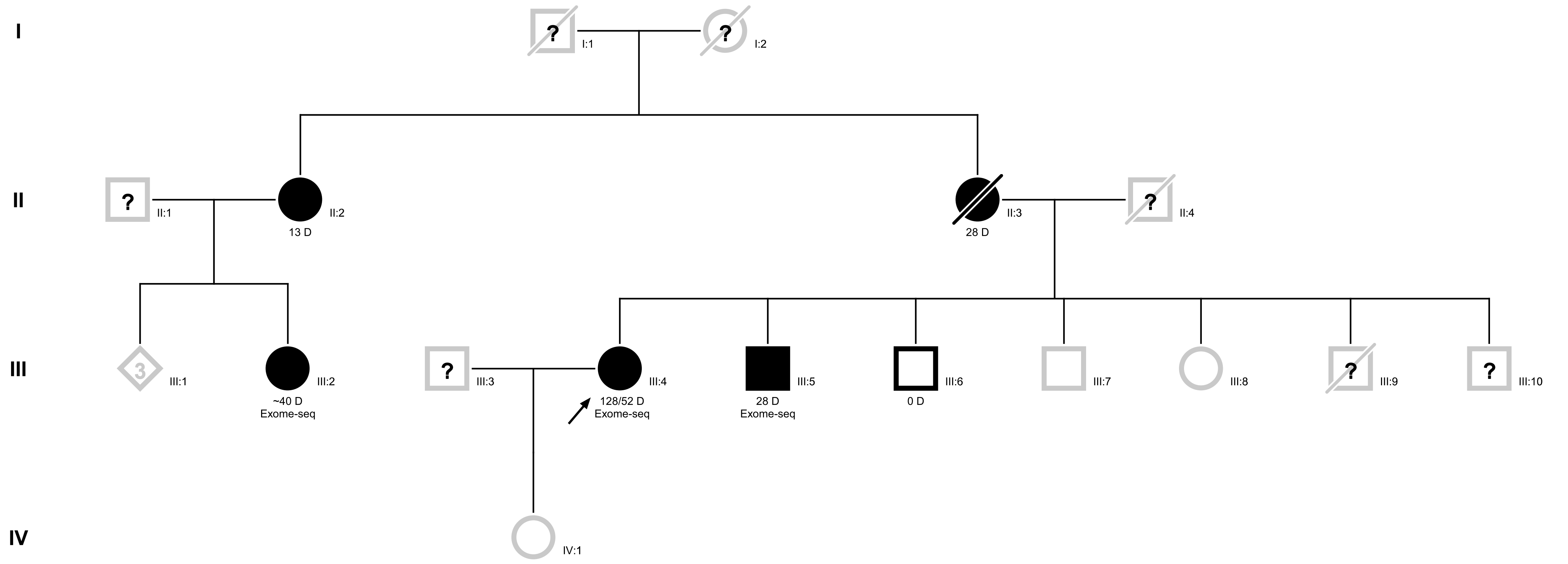
PC PAL
I
II
III
IV
?
I:1
?
I:2
?
II:1
II:2
13 D
II:3
28 D
?
II:4
3
III:1
III:2
~40 D
Exome-seq
?
III:3
III:4
128/52 D
Exome-seq
III:5
28 D
Exome-seq
III:6
0 D
III:7
III:8
?
III:9
?
III:10
IV:1

Family U

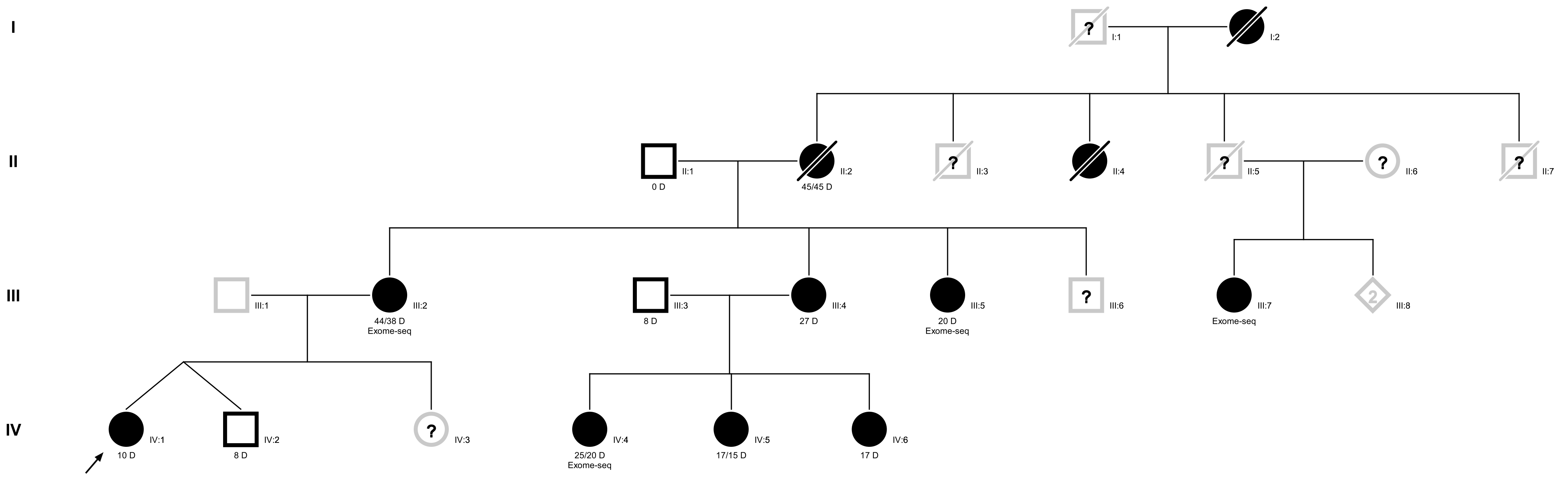
I
II
III
IV
?
I:1
I:2
II:1
0 D
II:2
45/45 D
?
II:3
II:4
?
II:5
?
II:6
?
II:7
III:1
III:2
44/38 D
Exome-seq
III:3
8 D
III:4
27 D
III:5
20 D
Exome-seq
?
III:6
III:7
Exome-seq
2
III:8
IV:1
10 D
IV:2
8 D
?
IV:3
IV:4
25/20 D
Exome-seq
IV:5
17/15 D
IV:6
17 D

Family V

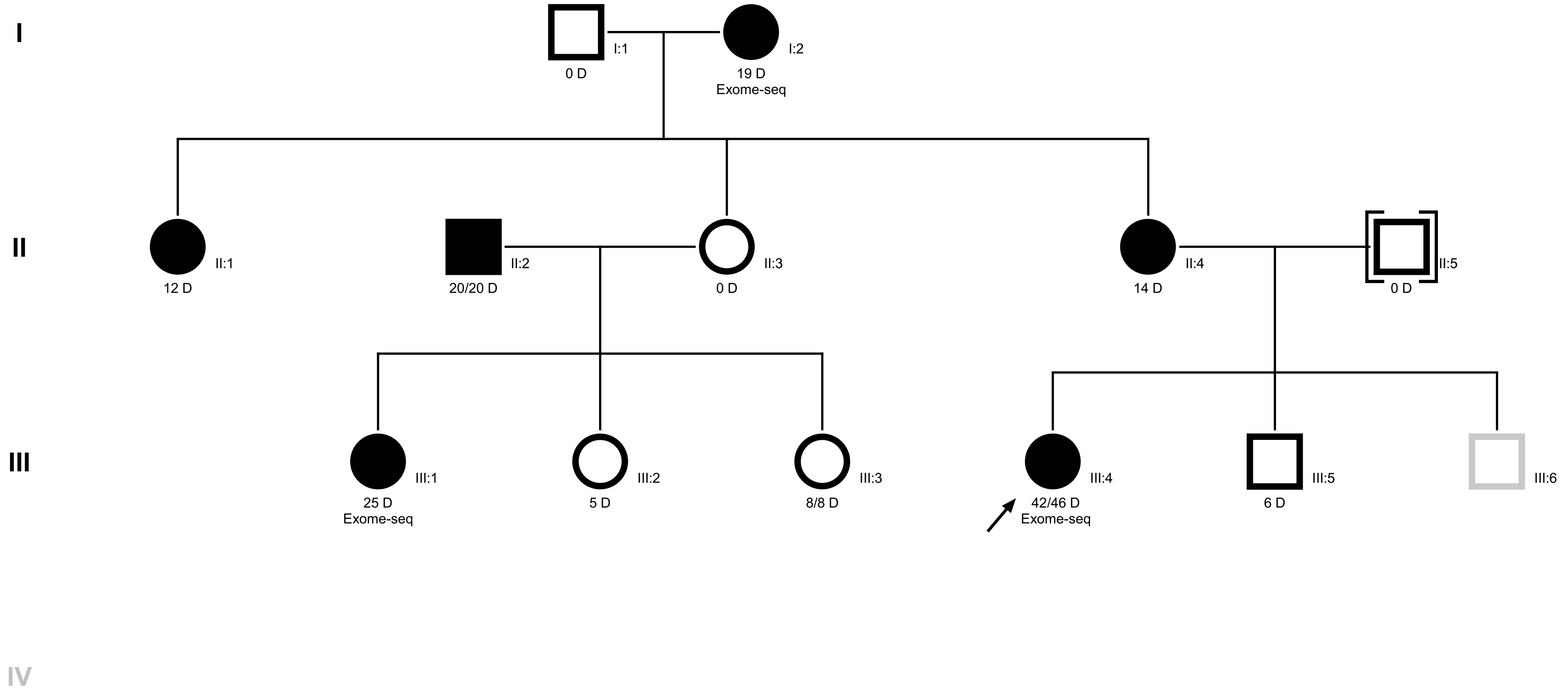

PC PAL
I
II
III
IV
I:1
0 D
I:2
19 D
Exome-seq
II:1
12 D
II:2
20/20 D
II:3
0 D
II:4
14 D
II:5
0 D
III:1
25 D
Exome-seq
III:2
5 D
III:3
8/8 D
III:4
42/46 D
Exome-seq
III:5
6 D
III:6

Family W

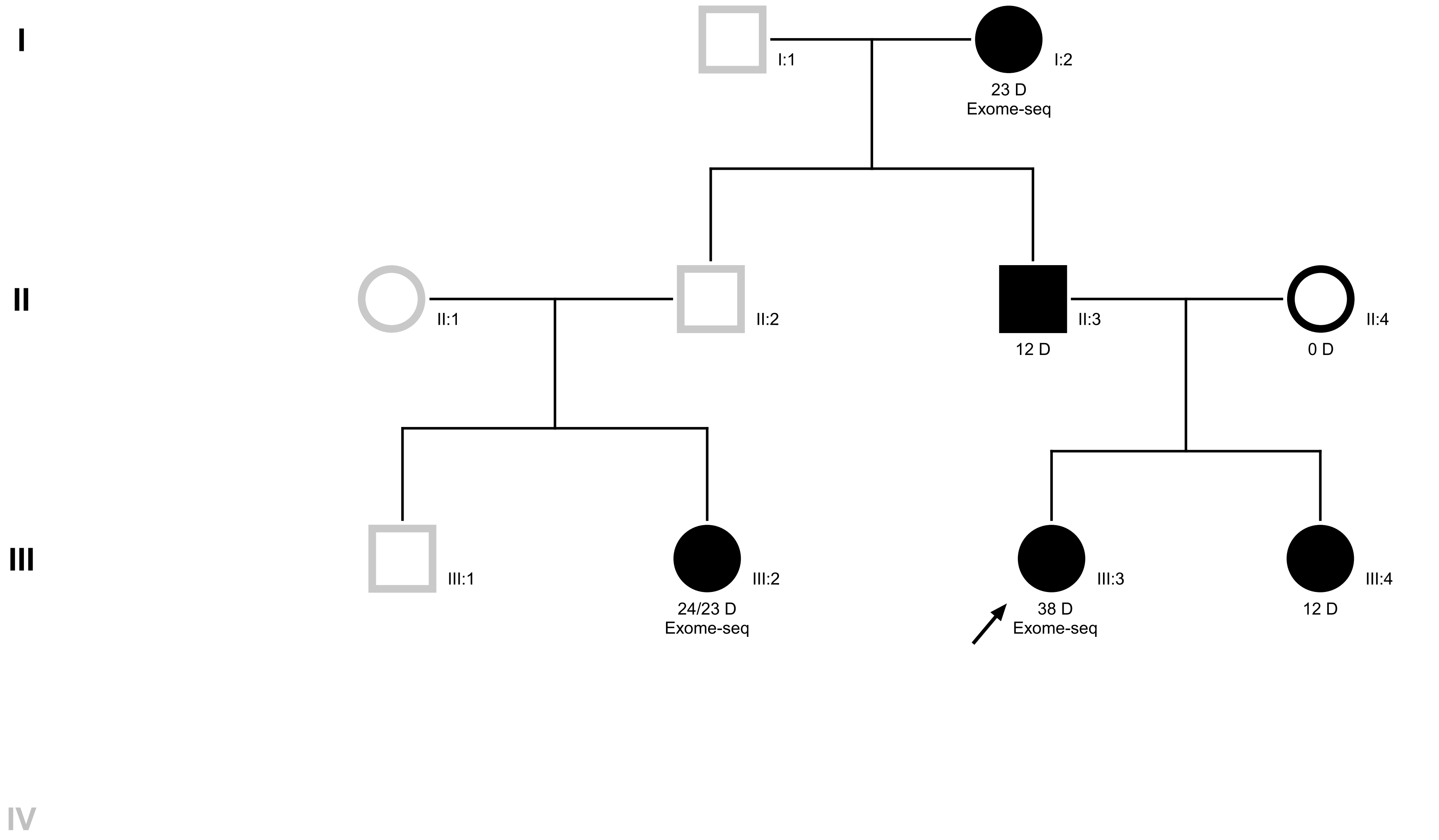

I
II
III
IV
I:1
I:2
23 D
Exome-seq
II:1
II:2
II:3
12 D
II:4
0 D
III:1
III:2
24/23 D
Exome-seq
III:3
38 D
Exome-seq
III:4
12 D
