# Supplementary material for: Whole Exome Sequencing of 23 Multigeneration Idiopathic Scoliosis Families Reveals Enrichments in Cytoskeletal Variants, Suggests Highly Polygenic Disease
Source: Genes (Basel). 2021 Jun 16;12(6):922. doi: 10.3390/genes12060922 (PMC8235452; doi:10.3390/genes12060922)
Supplement: Supplementary file 1 [file genes-12-00922-s001.zip › genes-1246102-supplementary-final/File S2.pdf]

<u>Supplemental File 2</u>

Supplemental Table 1: Clinical characteristics of probands and family members whose samples were used for exome sequencing. Probands are indicated by an * beside their pedigree ID (not all probands were exome sequenced). Pedigrees are provided in **Supplemental File 1**. Curve pattern abbreviations are as follows: RT or LT= Right/Left thoracic, RTL or LTL= Right/Left Thoracolumbar RL or LL= Right/Left Lumbar. Scoliosis curves are provided in degrees along with the vertebral site of their curve apex. Subjects with double curves have measurements and apex sites for both major and minor curves provided.

Pedigree ID	Sex	Relationship to Proband	Curve Pattern	Curve (Apex)	Surgery/Bracing
A-III:1	F	Sister	RT	18° (T8)	No
A-II:2	M	Uncle (maternal)	RT	35° (T8)	No
A-III:3*	F	Self	RT	50° (T8)	Surgery
A-I:2	F	Grandmother (maternal)	RT	20° (T8)	No
A-II:4	F	Aunt (maternal)	Unknown	Unknown curvature	No
B-III:5*	F	Self	RT/ LTL	36/54° (T7/T12-L1)	Brace/Surgery
B-I:2	F	Grandmother (paternal)	RTL	24° (T12-L1)	No
B-II:3	F	Aunt (paternal)	RT	50° (T8)	Surgery
B-III:1	M	Cousin (paternal)	RT	24°	No
C-III:2	F	Niece	RT	35° (T8)	No
C-III:3	M	Son	RT/ LL	62/48° (T8/L2)	Surgery
C-III:1	F	Niece	RT	34° (T8)	No
D-III:2*	F	Self	RT/ LL	50/35° (T8/L2)	Brace/Surgery
D-I:1	M	Grandfather (maternal)	RT	25° (T8)	No
D-III:4	F	Sister	Unknown	38°	Surgery
D-IV:3	F	Niece	Unknown	Unknown curvature	Brace/Surgery
E-II:2*	F	Self	RT/ LL	70/78° (T8/L2)	No
E-I:2	F	Mother	LTL	45° (L1)	No
E-III:4	F	Niece	Unknown	50°	No
E-III:5	F	Niece	RT	15° (T8)	No
F-III:1	M	Uncle (maternal)	LL	14° (L2)	No
F-IV:1*	F	Self	LT/ RT	36/51° (T4/T9)	Surgery
F-III:6	F	Cousin once removed (maternal)	Unknown	22°	No
F-IV:3	M	Second cousin	Unknown	20°	No
G-III:1	F	Daughter	Unknown	32/25°	Brace
G-III:3	F	Daughter	RT/ LTL	30/25° (T8/L1)	Brace
G-III:2	M	Son	LL	19° (L2)	No
G-I:2	F	Mother	Unknown	25°	No
G-II:1	M	Brother	Unknown	25°	No
H-II:6	F	Sister	LL	47° (L2)	Surgery
H-I:2	F	Mother	RT	15° (T8)	No
H-III:4	F	Daughter	LTL	10° (L1)	Brace
H-III:3	F	Niece	RTL	20° (T12)	No
I-II:8*	F	Self	RT/ LL	82/35° (T8/L2)	Surgery
I-III:5	F	Niece	Unknown	60°	Surgery
I-IV:1	F	Grand Niece	RT/ LTL	14/18° (T8/L2)	No
J-III:4*	F	Self	RT/ LL	35° (T8)	Brace/Surgery
J-III:2	F	Cousin (maternal)	Unknown	50°	Brace/Surgery
J-III:3	M	Brother	RT	14° (T11)	No
J-III:5	F	Sister	RT/ LTL	18/13° (T8/L1)	No
K-II:2	F	Sister	Unknown	15°	No

K-II:1	F	Sister	RT/ LL	20/50° (T8/L2)	Surgery
K-III:1	M	Son	LT	50° (T8)	Brace
K-III:3	M	Son	RT/ LL	30/21° (T9/L3)	Brace
L-II:3*	F	Self	RT/ LL	68/53° (T9/L2)	Surgery
L-IV:2	F	Grand Niece	LTL	20° (T12)	Brace
L-IV:3	F	Grand Niece	LL	30°	No
M-III:3	F	Niece	RT	35° (T8)	No
M-I:2	F	Mother	RL	30° (L3)	No
M-II:6*	F	Self	LL	61° (L1-L2)	Surgery
M-III:5	F	Niece	RT	18° (T9-T10)	No
N-III:2	F	Daughter	RT/ LTL	30/27° (T7/T12-L1)	Brace
N-III:6	M	Nephew	LT/ RT	13/11° (T2-T3/T5-T6)	No
N-III:1	M	Son	RT	20° (T6)	No
O-IV:1	F	Second cousin	RT/ LL	50/35° (T8-T9/L2)	Brace/Surgery
O-IV:3	F	Sister	RT	57° (T9-T10)	Brace/Surgery
O-IV:2*	F	Self	RT/ LTL	20/16° (T8-T9/L1)	Brace
P-II:2*	F	Self	RT/ LL	82/67° (T9/L2)	No
P-III:2	F	Niece	LT/ RT	36/16° (T3/T8-T9)	No
P-II:6	F	Sister	LL	17° (L1-L2)	No
P-II:7	F	Sister	Unknown	50°	Brace/Surgery
Q-III:5*	F	Self	RT/ LL	70/66° (T8/L2)	Surgery
Q-I:2	F	Grandmother (paternal)	LTL	14° (T12-L1)	No
Q-III:2	M	Cousin (paternal)	LL	15° (L2-L3)	No
R-III:1	M	Son	Unknown	35°	No
R-II:2*	F	Self	RT/ LTL	37/47° (T8/L1-L2)	No
R-III:2	M	Son	RT	22° (T8-T9)	No
R-III:5	M	Son	RT	48° (T9-T10)	Brace/Surgery
R-IV:2	M	Grandson	RT/ LTL	32/24° (T9/L2)	No
S-II:2*	F	Self	RT/ LL	45/56° (T8/L2)	Surgery
S-III:2	F	Niece	LT/ RT	20/12° (T4-T5/T10)	No
S-III:3	F	Niece	LTL	22° (T12-L1)	No
S-I:2	F	Mother	RL	65° (L2)	No
T-III:4*	F	Self	RT/ LL	128/52° (T6/L1-L2)	No
T-III:5	M	Brother	RT	28° (T9-T10)	No
T-III:2	F	Cousin (maternal)	RTL	40°	Brace
U-III:2	F	Mother	RT/ LL	44/38° (T7-T8/L2)	Surgery
U-IV:4	F	Cousin (maternal)	RT/ LTL	15/15° (T8/L1)	No
U-III:5	F	Aunt (maternal)	Unknown	20°	No
U-III:7	F	Cousin once removed (maternal)	Unknown	Unknown curvature	No
V-I:2	F	Grandmother (maternal)	LL	19° (L2-L3)	No
V-III:4*	F	Self	RT/ LTL	42/46° (T8/L1-L2)	Brace
V-III:1	F	Cousin (maternal)	RTL	25° (L1)	No
W-III:3*	F	Self	Unknown	38°	No
W-I:2	F	Grandmother (paternal)	Unknown	23°	No
W-III:2	F	Cousin (paternal)	RT/ LL	24/23° (T9/L2-L3)	No

Supplemental Table 2: Enriched cytobands from MAF<0.05 input gene list (n= 1161) using DAVID, as described in the methods.

Term	Count	%	*p* Value	Genes	Fold Enrichment	Bonferroni
16p13.3	23	2.0070	0.0001	*SRRM2, TRAP1, OR2C1, TPSB2, TPSG1, IFT140, ABCA3, TSC2, KREMEN2, PPL, RPL3L, VASN, CACNA1H, SSTR5, LMF1, RAB26, EME2, E4F1, CORO7, FLYWCH1, RGS11, PRR25, MSRB1*	2.6930	0.0350
5q35.2	7	0.6108	0.0016	*RNF44, UNC5A, GPRIN1, FAM153B, CDHR2, HMP19, THOC3*	5.4784	0.6443
19q13.42	8	0.6981	0.0043	*ZSCAN5B, TARM1, SSC5D, RDH13, KIR2DL1, TMC4, FAM71E2, OSCAR*	3.9003	0.9409
2q37.3	9	0.7853	0.0054	*FARP2, RTP5, PRR21, NEU4, AQP12A, HDLBP, KIF1A, SCLY, BOK*	3.3458	0.9727
16q24.3	7	0.6108	0.0093	*ANKRD11, FANCA, PIEZO1, ACSF3, SLC22A31, RNF166, GAS8*	3.8552	0.9979
15q11.2-q21.3	3	0.2618	0.0105	*JMJD7-PLA2G4B, PLA2G4B, NDUFAF1*	17.8441	0.9991
3q13.2	5	0.4363	0.0136	*TMPRSS7, ABHD10, BOC, SIDT1, SLC9C1*	5.3107	0.9999
3q29	9	0.7853	0.0156	*LRRC15, TM4SF19, DLG1, TMEM44, TNK2, ATP13A4, ATP13A3, XXYLT1, MUC4*	2.7881	1.0000
19p13.3	16	1.3962	0.0201	*CFD, SMIM24, KDM4B, MLLT1, ABCA7, ZNF77, MFSD12, ADAMTS10, PCSK4, CNN2, GZMM, PLIN4, TRIP10, FSD1, APBA3, SLC25A23*	1.9343	1.0000
19q13.4	8	0.6981	0.0229	*LENG1, PEG3, KIR3DL1, LILRB1, KIR2DL1, PTPRH, GP6, ZIM2*	2.8324	1.0000
1q44	9	0.7853	0.0307	*OR2T3, PGBD2, OR1C1, OR14K1, OR2T29, EFCAB2, CEP170, OR2T7, OR14A2*	2.4556	1.0000
1q21	9	0.7853	0.0322	*SPTA1, PIAS3, FAM189B, FCRL2, PGLYRP4, SETDB1, ARNT, ANXA9, MCL1*	2.4333	1.0000
10q26	4	0.3490	0.0362	*PTPRE, CHST15, TACC2, EIF3A*	5.4073	1.0000
19q13.1	6	0.5236	0.0422	*GPI, FCGBP, ITPKC, NPHS1, ZNF527, FFAR3*	3.1305	1.0000
11q13	9	0.7853	0.0496	*ESRRA, PLCB3, NDUFS8, NUMA1, SLCO2B1, FOLR3, ZFPL1, SIPA1, SPTBN2*	2.2305	1.0000
10q24.33	3	0.2618	0.0507	*PDCD11, SH3PXD2A, INA*	8.1109	1.0000
2p24-p23	2	0.1745	0.0661	*APOB, MATN3*	29.7401	1.0000
10p12.33	3	0.2618	0.0688	*MRC1, SLC39A12, MALRD1*	6.8631	1.0000
1q23.1	5	0.4363	0.0771	*RGS5, OR6Y1, OR10T2, OR6P1, NES*	3.0979	1.0000
1q32.1	8	0.6981	0.0776	*DSTYK, SLC45A3, ATP2B4, PLEKHA6, SRGAP2, LAX1, IGFN1, CAMSAP2*	2.1629	1.0000
1q21.3	7	0.6108	0.0932	*KPRP, FLG, ATP8B2, KCNN3, SLC27A3, LCE1E, LCE2D*	2.2385	1.0000

Supplemental Table 3: We genotyped specific variants in additional affected and unaffected family members. Variants were selected for validation that were present in multiple families and appeared within enriched functional categories (cytoskeleton and/or extracellular matrix GO Terms). Families containing these variants also had to have additional family members that provided a DNA sample. Ensembl HGVS notation is provided for the splice site variant, indicated with a *. Families indicated with a “2018-” were previously exome sequenced using a similar pipeline and published by our laboratory (Baschal et al., *G3* 2018, PMC6071588).

					Genotyping in additional family members (not exome sequenced)		
**Gene**	**GO Term(s)**	**Family**	**WES *n* AIS**	**Variant (HGVS)**	**AIS with variant**	**Unaffected with variant**	**Segregated with AIS phenotype?**
ANKRD11	*Plasma membrane, cytoplasm*	C	3	ANKRD11:NM_001256183:exon9:c.C6176A:p.P2059H,ANKRD11:NM_013275:exon9:c.C6176A:p.P2059H,ANKRD11:NM_001256182:exon10:c.C6176A:p.P2059H	4/4	2/3	*Partially*
		H	4	ANKRD11:NM_001256183:exon9:c.C7022T:p.A2341V,ANKRD11:NM_013275:exon9:c.C7022T:p.A2341V,ANKRD11:NM_001256182:exon10:c.C7022T:p.A2341V	0/6	0/3	*No*
		S	4	ANKRD11:NM_001256183:exon9:c.C7022T:p.A2341V,ANKRD11:NM_013275:exon9:c.C7022T:p.A2341V,ANKRD11:NM_001256182:exon10:c.C7022T:p.A2341V	0/2	0/3	*No*
		2018-E	3	ANKRD11:NM_001256183:exon9:c.C7106T:p.A2369V,ANKRD11:NM_013275:exon9:c.C7106T:p.A2369V,ANKRD11:NM_001256182:exon10:c.C7106T:p.A2369V	0/4	0/3	*No*
		2018-E	3	ANKRD11:NM_001256183:exon9:c.C7061A:p.P2354H,ANKRD11:NM_013275:exon9:c.C7061A:p.P2354H,ANKRD11:NM_001256182:exon10:c.C7061A:p.P2354H	0/4	0/3	*No*
COL21A1	*Proteinaceous extracellular matrix, collagen trimer*	P	4	COL21A1:NM_030820:exon16:c.C1748G:p.P583R	1/4	1/4	*No*
		S	4	COL21A1:NM_030820:exon28:c.T2462C:p.L821P	NA	2/3	*No*
COL6A5	*Collagen trimer, collagen catabolic process, cell adhesion*	*2018-B*	3	COL6A5:NM_001278298:exon38:c.A7300G:p.K2434E,COL6A5:NM_153264:exon38:c.A7300G:p.K2434E	2/5	3/7	*No*
		C	3	COL6A5:NM_001278298:exon40:c.T7580C:p.L2527P	0/3	2/3	*No*
		H	4	COL6A5:NM_001278298:exon3:c.T167C:p.M56T,COL6A5:NM_153264:exon3:c.T167C:p.M56T	3/5	1/3	*Partially*
FGD1	*Cytoplasm, cytoskeleton, actin cytoskeleton regulation*	E	4	FGD1:NM_004463:exon3:c.G515C:p.R172P	0/4	0/3	*No*
		J	4	FGD1:NM_004463:exon3:c.G515C:p.R172P	0/2	N/A	*No*
NPHP4	*Cytoskeleton, centrosome, actin cytoskeleton organization*	*2018-A*	4	NPHP4:NM_001291594:exon13:c.G683A:p.R228H,NPHP4:NM_001291593:exon14:c.G680A:p.R227H,NPHP4:NM_015102:exon17:c.G2219A:p.R740H	2/4	1/5	*Partially*
		O	3	*NPHP4:ENST00000378156:exon20:c.2818-2A>T:NA	NA	1/2	*Unknown*
		W	3	NPHP4:NM_001291594:exon15:c.C1006T:p.R336W,NPHP4:NM_001291593:exon16:c.C1003T:p.R335W,NPHP4:NM_015102:exon19:c.C2542T:p.R848W	1/2	3/5	*No*
OBSCN	*Cell-cell junction, integral*	C	3	OBSCN:NM_001098623:exon16:c.G4801A:p.A1601T,OBSCN:NM_052843:exon16:c.G4801A:p.A1	2/4	3/3	*No*

	component of plasma membrane, Z disc			601T,OBSCN:NM_001271223:exon18:c.G5353A:p.A1785T			
		C	3	OBSCN:NM_001098623:exon53:c.C13984T:p.R4662C,OBSCN:NM_052843:exon53:c.C13984T:p.R4662C,OBSCN:NM_001271223:exon64:c.C16855T:p.R5619C	2/4	3/3	*No*
		J	4	OBSCN:NM_001098623:exon11:c.A3473T:p.H1158L,OBSCN:NM_052843:exon11:c.A3473T:p.H1158L,OBSCN:NM_001271223:exon12:c.A3749T:p.H1250L	0/1	NA	*Unknown*
		U	3	OBSCN:NM_001098623:exon22:c.G6373T:p.A2125S,OBSCN:NM_052843:exon22:c.G6373T:p.A2125S,OBSCN:NM_001271223:exon26:c.G7498T:p.A2500S	3/4	1/5	*Yes*
TNXB	*Extracellular matrix structural constituent, actin cytoskeleton organization, proteinaceous extracellular matrix, cell adhesion*	*2018-A*	4	TNXB:NM_032470:exon8:c.T1298C:p.M433T,TNXB:NM_019105:exon39:c.T12005C:p.M4002T	4/4	3/5	*Partially*
		G	5	TNXB:NM_019105:exon24:c.C8192G:p.P2731R	3/3	0/1	*Yes*
		S	4	TNXB:NM_019105:exon12:c.G4444A:p.V1482M	NA	1/3	*Unknown*
CTNNA3	*Adherens junction, cell adhesion, cytoplasm, cytoskeleton*	I	3	CTNNA3:NM_001127384:exon5:c.T478A:p.S160T,CTNNA3:NM_013266:exon5:c.T478A:p.S160T,CTNNA3:NM_001291133:exon6:c.T514A:p.S172T	1/1	3/5	*Yes*
		W	3	CTNNA3:NM_001127384:exon12:c.C1603T:p.R535C,CTNNA3:NM_013266:exon12:c.C1603T:p.R535C	2/2	1/5	*Yes*
NTRK1	*Nerve growth factor signaling pathway, axon guidance, positive regulation of neuron projection development, plasma membrane*	C	3	NTRK1:NM_001012331:exon6:c.G631A:p.V211M,NTRK1:NM_002529:exon6:c.G631A:p.V211M,NTRK1:NM_001007792:exon7:c.G541A:p.V181M	3/4	1/3	*Yes*
		T	3	NTRK1:NM_001012331:exon14:c.C1792T:p.H598Y,NTRK1:NM_001007792:exon15:c.C1702T:p.H568Y,NTRK1:NM_002529:exon15:c.C1810T:p.H604Y	2/2	0/4	*Yes*
		T	3	NTRK1:NM_001012331:exon14:c.G1820T:p.G607V,NTRK1:NM_001007792:exon15:c.G1730T:p.G577V,NTRK1:NM_002529:exon15:c.G1838T:p.G613V	2/2	0/4	*Yes*
PDE4DIP	*Actin cytoskeleton organization, cytoplasm, centrosome*	T	3	PDE4DIP:NM_001002811:exon1:c.A322C:p.I108L	2/2	0/4	*Yes*
		W	3	PDE4DIP:NM_001002811:exon1:c.C317T:p.A106V	4/4	4/5	*No*

Supplemental Table 4: Complete list of enriched Gene Ontology terms p< 0.01 using DAVID. Input gene list was the combined Minor Allele Frequency (MAF) <0.05 gene list from all families (input n= 1161). Default values in DAVID were used.

**Category**	**Term**	**Count**	**%**	**PValue**	***Genes***	**Fold Enrichment**	**FDR**
GOTERM_MF_DIRECT	GO:0005089~Rho guanyl-nucleotide exchange factor activity	15	1.31	0.00	*FARP2, PLEKHG4, PLEKHG1, PLEKHG2, ARHGEF28, KALRN, FGD1, AKAP13, OBSCN, ALS2, EPS8L2, ARHGEF40, DOCK1, MCF2L2, SOS2*	3.35	0.12
GOTERM_BP_DIRECT	GO:0007411~axon guidance	23	2.01	0.00	*VASP, NTRK1, EPHA5, SPTBN5, TENM2, UNC5A, BDNF, PTPRO, ANK3, SEMA3F, GLI3, ROBO1, SPTA1, KIF26A, GPC1, FEZ1, BOC, DVL1, APBB2, NCAM1, SLIT2, MATN2, SPTBN2*	2.47	0.27
GOTERM_CC_DIRECT	GO:0005874~microtubule	36	3.14	0.00	*INVS, DNAH1, DNAH7, TUBAL3, DCTN1, DNAH6, IQGAP1, CAMSAP2, GOLGA2, TEKT1, DVL1, KIF13B, TEKT2, CEP170, KIF21B, FSD1, KIF1A, PCNT, DYNC2H1, DNAH14, MTUS2, KCNAB2, SHROOM1, HAUS5, EML2, DLG1, SYNJ1, KATNAL2, INCENP, KIF26A, KIFC1, FEZ1, EHHADH, TTLL11, EIF3A, GAS8*	1.98	0.09
GOTERM_BP_DIRECT	GO:0035023~regulation of Rho protein signal transduction	15	1.31	0.00	*FARP2, PLEKHG4, PLEKHG1, PLEKHG2, ARHGEF28, KALRN, FGD1, AKAP13, OBSCN, ALS2, DLC1, EPS8L2, ARHGEF40, MCF2L2, SOS2*	3.16	0.27
GOTERM_BP_DIRECT	GO:0043547~positive regulation of GTPase activity	55	4.80	0.00	*RGSL1, DOCK8, KNDC1, SIPA1L3, RPGR, RGS5, AKAP13, RGS3, PLCE1, ARHGEF40, SBF1, MCF2L2, RGS6, AGFG2, PLEKHG1, PTGIR, VPS9D1, PLEKHG2, TSC2, NRG1, ARAP1, GMIP, ACAP1, OBSCN, ALS2, MADD, RAPGEF2, RIN1, DOCK1, SOS2, RGS18, SPTBN5, ARHGEF28, AGAP1, IQGAP1, RASAL3, KALRN, FGD1, SPTA1, EPS8L2, NCAM1, SRGAP2, SRGAP1, SPTBN2, NTRK1, FARP2, STARD13, EGF, DAB2IP, ARHGAP24, DLC1, TRIP10, RGS11, SIPA1, LAMTOR5*	1.66	0.27
GOTERM_BP_DIRECT	GO:0032091~negative regulation of protein binding	12	1.05	0.00	*GOLGA2, EPB41L5, FRMD7, DVL1, CCM2L, DTNBP1, TMC8, CAMK1, PPARA, NES, SORL1, DACT1*	3.59	0.32
GOTERM_MF_DIRECT	GO:0019829~cation-transporting ATPase activity	5	0.44	0.00	*ATP7B, ATP8A1, ATP13A4, ATP13A2, ATP13A3*	10.73	0.28
GOTERM_CC_DIRECT	GO:0036057~slit diaphragm	5	0.44	0.00	*TRPC6, KIRREL2, NPHS1, MAGI2, IQGAP1*	10.66	0.17
GOTERM_MF_DIRECT	GO:0008514~organic anion transmembrane transporter activity	6	0.52	0.00	*SLCO2B1, SLCO1A2, SLCO1B3, SLC22A31, MFSD10, SLC22A11*	7.36	0.28
GOTERM_CC_DIRECT	GO:0005882~intermediate filament	17	1.48	0.00	*FLG, DSP, KRTAP13-4, KRT2, KRTAP26-1, KRTAP27-1, KRT79, KRT10, GFAP, KRT28, KRT37, PKP2, KRTAP6-1, SYNC, NES, KRT6A, PRPH*	2.57	0.17
GOTERM_CC_DIRECT	GO:0030018~Z disc	17	1.48	0.00	*PPP1R12A, SYNPO2, AHNAK2, ATP2B4, SLC4A1, NEB, ANK3, ADRA1A, IGFN1, RYR3, MYPN, TTN, OBSCN, HRC, PDE4B, SYNC, CRYAB*	2.46	0.21
GOTERM_MF_DIRECT	GO:0005096~GTPase activator activity	30	2.62	0.00	*RGS18, RGSL1, AGAP1, IQGAP1, RASAL3, KALRN, SIPA1L3, SYDE2, RGS5, RGS3, SRGAP2, SRGAP1, AGFG2, RGS6, STARD13, VPS9D1, DAB2IP, TSC2, ARAP1, GMIP, ARHGAP24, ACAP1, DLC1, TRIP10, RAPGEF2, RGS11, RIN1, DOCK1, SIPA1, TBC1D26*	1.85	0.45
GOTERM_CC_DIRECT	GO:0005819~spindle	17	1.48	0.00	*INVS, DIDO1, SPAG8, NUMA1, DCTN1, DCTN3, HEPACAM2, HAUS5, EML2, INCENP, KIFC1, NUP85, CLTCL1, ANKRD53, CEP170, E4F1, KBTBD8*	2.40	0.22
GOTERM_BP_DIRECT	GO:0051056~regulation of small GTPase mediated signal transduction	18	1.57	0.00	*STARD13, PLEKHG2, TSC2, ARAP1, GMIP, KALRN, SIPA1L3, FGD1, ARHGAP24, SYDE2, AKAP13, OBSCN, DLC1, TRIP10, SRGAP2, SRGAP1, SOS2, SIPA1*	2.29	1.00
GOTERM_BP_DIRECT	GO:0042060~wound healing	13	1.13	0.00	*DSP, ALOX15, CCM2L, FN1, NRG1, SCRIB, GLI3, DCBLD2, CNN2, NLRP6, PTK7, PPARA, KRT6A*	2.77	1.00
GOTERM_CC_DIRECT	GO:0005737~cytoplasm	347	30.28	0.00	*RPL5, ANKLE1, MTRR, HDAC10, RGSL1, ABCA12, BACH2, WDR87, ENDOV, TBK1, CYP2D7, MPRIP, PPP4R2, C7ORF31, FAM65C, PLCE1, TRIM26, RTTN, KIF21B, ADGB, SDS, BSX, KRT2, MTUS2, AFAP1, SCRIB, LCE1E, MAPK8IP2, EML2, PPP1R3G, INPP4B, KATNAL2, TEP1, FEZ1, ZSCAN26, WDPCP, TTLL11, FLYWCH1, ALPK2, PFN1, RIN1, TRIB2, KPRP, SPTBN5, PARPBP, DHX8, PCDH15, C1ORF198, AGAP1, XPC, IQGAP1, EVPL, EPB41L5, TESPA1, SPATA5L1, HLCS, XRRA1, DHX35, EPB41L2, AFMID, PLEK2, KCNN3, APOB, SPTBN2,*	1.13	0.27

					VPS16, VASP, SRMS, SETDB1, PMM1, DNAH14, NFATC3, THAP11, BPIFB3, NDUFAF1, CPEB2, INVS, GPI, TENM1, ZCCHC11, MTMR12, TENM4, STAB2, FMN1, IKBKAP, STON2, SERPINA9, ECSIT, PTPRH, METTL11B, PFAS, LYST, LITAF, GLI3, ROBO1, AKAP12, AKAP13, TRIM6, DSTYK, KRT28, SH3PXD2A, KIF13B, TNKS1BP1, CD34, LRRCC1, APOBEC1, CCDC116, RING1, IFT140, DUSP1, PARP2, STRBP, TMC8, BTN3A3, PPHLN1, PLIN4, RARA, STRAP, RAPGEF2, UMPS, CRYAB, CD44, LRRC15, AHCY, AHNAK, ADCY2, AKAP3, FGD1, LCE2D, PRSS42, CAMKK2, LIMA1, ARVCF, ANKRD53, APBB2, CTNNA3, MAP4K5, PCNT, APOL3, RUNX1T1, FARP2, WWOX, SVIL, CASP8AP2, RFTN1, MBD2, DAB2IP, LILRB1, VWA3B, KRT10, FMO5, PSMB9, FAM200A, MYBBP1A, NEDD4, DLC1, TTC3, ALMS1, TRIP10, FAS, FILIP1, TACC2, CAMK1, INHA, DFNA5, TJP2, MPHOSPH6, NEUROG3, GPHB5, ERCC6L, MROH2B, ZBTB25, NCF2, ATN1, HDLBP, UBE3B, CDC14A, PARK2, SYDE2, STK10, RGS5, HELB, RGS3, PPIP5K1, TEKT1, TEKT2, SVEP1, TGM6, NEK3, ARHGEF40, ZNF322, PIAS3, USP8, RNF44, AGTPBP1, MAGI2, TSC2, FRMD4A, CHD1L, KRT74, LAX1, SDCBP2, ZNF318, MADD, ADAM8, ELMO3, SULT6B1, HOMEZ, DRC7, BHLHB9, TSACC, ANKRD11, SPATA2, FOXO3, RASAL3, CAMSAP2, KIAA0556, FBXO40, ATXN3, NPAT, ORC4, ATXN1, PDZD2, STK36, PLXNA1, E4F1, RHPN2, SRGAP2, CCDC141, SRGAP1, MCL1, FAM83A, ZFHX3, COA1, SMURF2, BDNF, EPHX2, TNK2, ECD, FANCA, KLHL21, SSC5D, FBXO34, DNAJC13, TDRD6, MCC, EIF2S1, GFAP, PTPRE, DLG1, NASP, UBXN11, NACA, MAP3K19, BOD1L2, ACO1, NF2, NES, DIDO1, ALAS1, DYRK2, NUMA1, USP32, MTCL1, EFCAB13, MCM10, GPHN, TTC28, SYNE1, HEBP1, PIDD1, SGCD, SIN3B, ZIC3, METTL10, MYADML2, CEP170, PRKG1, DACT1, BOK, GET4, TRIM67, DSP, OVGP1, TTC37, PPP1R12A, TRPC6, TBP, VPS13C, PPP2R5B, ARNT, NRG1, KCNAB2, SHROOM1, MUC5AC, TFDP3, EEF1D, EHHADH, BPI, GNB1L, CFAP54, DOCK1, FAN1, RGS18, KDM3A, DNAH1, DNAH7, TUBAL3, DCTN1, BRAT1, VPS4B, DNAH6, PALM3, MLLT1, PARD6B, DNPEP, KCTD20, ASB10, CLMN, ANKAR, RNF39, SH3BP4, NCAM1, EPS8L2, SLIT2, FSD1, NXF5, DCAF13, MDH1B, CDK11A, KDM4B, SPAG8, PPIL2, DYX1C1, USP21, MGA, AHNAK2, DTNBP1, TPRKB, PDE4DIP, SSRP1, PTPN13, CTCFL, NELL1, CROCC, RGS11, LPIN1, EIF3A		
GOTERM_MF_DIRECT	GO:0008236~serine-type peptidase activity	11	0.96	0.00	*CFD, ST14, TMPRSS7, TPSB2, GZMM, TPSG1, C1R, KLK5, PLG, PRSS3, HGFAC*	3.00	0.65
GOTERM_CC_DIRECT	GO:0016328~lateral plasma membrane	10	0.87	0.00	*CEACAM1, DLG1, KCNB1, MTCL1, ABCC6, PTPRO, DVL1, ANK3, IQGAP1, NKD2*	3.22	0.27
GOTERM_CC_DIRECT	GO:0005856~cytoskeleton	36	3.14	0.00	*TENM1, DRC7, MTCL1, EVPL, PPL, GPHN, FGD1, SYNE1, KIAA0556, CNN2, AKAP12, EPB41L5, SGCD, EPB41L2, TNKS1BP1, PLEK2, NPHP4, CTNNA3, TRIM67, DSP, VASP, FARP2, AFAP1, FRMD4A, KCNAB2, PTPN13, ARHGAP24, APBB1IP, UBXN11, FRMD7, TRIP10, FILIP1, NF2, RIN1, PFN1, TRIB2*	1.66	0.27
GOTERM_BP_DIRECT	GO:0015721~bile acid and bile salt transport	7	0.61	0.00	*SLC10A4, CEACAM1, SLC10A6, SLC10A2, SLCO2B1, SLCO1A2, SLCO1B3*	4.42	1.00

Family A

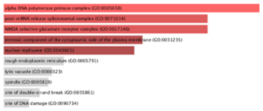

Cytoskeletal genes: VASP, KIFC1*

Family B

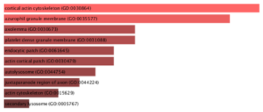

Cytoskeletal genes: FBN2*

Family C

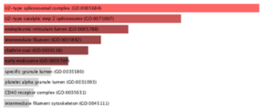

Cytoskeletal genes: NES, NEB, SLC4A1,

Family D

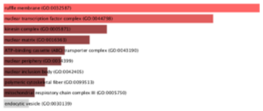

Cytoskeletal genes: LIMA1, MSRB1, ZNF286A, TENM1, KIF1A*, KIF26A*

Family E

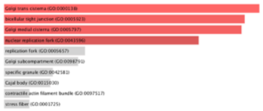

Cytoskeletal genes: SYNPO2,

Family F

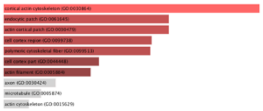

Cytoskeletal genes: EPB41L2,

Family G

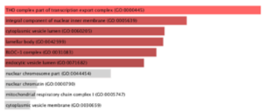

Cytoskeletal genes: DTNBP1, AHNAK2

Family H

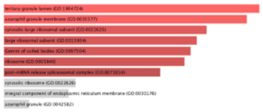

Cytoskeletal genes: CNN2, ZNF224, EML2

Family I

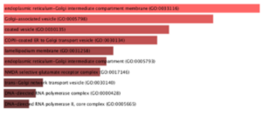

Cytoskeletal genes: RNF207, AFAP1, INA, SERPINA9

Family J

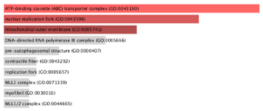

Cytoskeletal genes: IQGAP1, MPRIP,

Family K

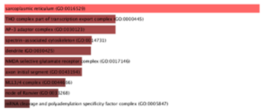

Cytoskeletal genes: EPB41L5, ANK3,

Family L

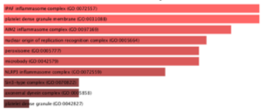

Cytoskeletal genes: KLHL21, DNAH1*, CEP83*

Family M

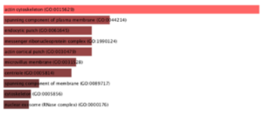

Cytoskeletal genes: CROCC, FILIP1, TACC2, RARA

Family N

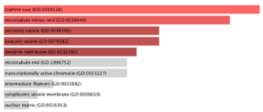

Cytoskeletal genes: SVIL, MPP2, ZNK234

Family O

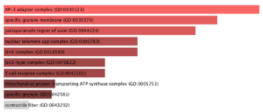

Cytoskeletal genes: LYST, PP1R12A, EVPL

Family P

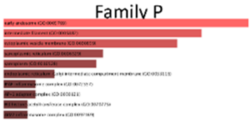

Cytoskeletal genes: SCNN1D, AHNAK2, NF2, DNAH7*, CEP170*

Family Q

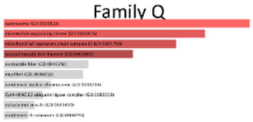

Cytoskeletal genes: CEP170*, RPGR*

Family R

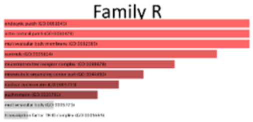

Cytoskeletal genes: CEP170*, CEP135*

Family S

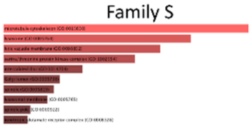

Cytoskeletal genes: CPTA1, DCTN1, HMMR, AHNAK, MTUS2

Family T

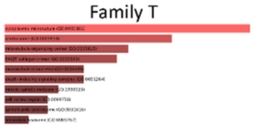

Cytoskeletal genes: AHNAK, MTUS2, CEP131

Family U

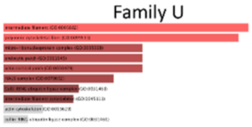

Cytoskeletal genes: KALRN, ABRA, MTCL1, DNAH6, KIF13B*

Family V

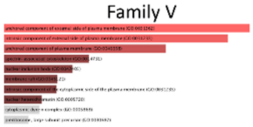

Cytoskeletal genes: SPTA1, SNF22, AHNAK, ZNF236

Family W

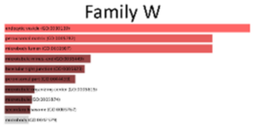

Cytoskeletal genes: NPHP4, KIF21B, DNAH14

***Supplemental Figure 1**:* Top enriched GO Cellular Component terms, by family, in order of descending p-value. Most families show an enrichment of cytoskeletal-related GO terms. Cytoskeletal genes containing variants within the family are also provided. Cytoskeletal genes were determined from the “cytoskeleton” GO term. Genes with a * were manually annotated.

Supplemental Table 5: PCR primer sequences for genotyping. Families indicated with "2018" were published previously by our group (Baschal et al., 2018, PMID: 29930198).

Gene	Family	Primer Direction	Primer Seq
ANKRD11	H, S, 2018-E	Forward	CCAGAGAGAGAAGGCAGTGG
		Reverse	GGGGATGTTTCCACCGTAGT
ANKRD11	C	Forward	AGACTTTCTTCCACGGGTTCC
		Reverse	CTCGGACCTCCTGCTGAAGT
COL21A1	P	Forward	TTAAGAATATGATGGTGCTAATGGA
		Reverse	CTGGACCTGCTGTGAGTTTG
COL21A1	S	Forward	TTTTAATCCTCTGACACCTGGAC
		Reverse	TCACTTTTTCCCCTATTTTAAGCA
COL6A5	2018-B	Forward	CACTCCACTGACCTCCACCT
		Reverse	AGAACCTGCCACATAGTAAACACTC
COL6A5	C	Forward	TATGAAGATTGGCAGGAAGGATT
		Reverse	CTGACTCCACACCAAGCATTT
COL6A5	H	Forward	CTGATATACACTTAACACCAGCACA
		Reverse	GCCTCTTCCACTTCATCCTC
FGD1	E, J	Forward	CTCTCCTGACTATCCCTTCCTGT
		Reverse	GTGCAGATTGGTGTTTGTGG
NPHP4	2018-A	Forward	ACGAGTGAACAAAGAAGGTCAAA
		Reverse	ACACGCCCAGAATAATAAACTCC
NPHP4	O	Forward	AGGAGTGGGAGAGAGAAGTCAAA
		Reverse	GCGATGCTTGTGGAGAAATG
NPHP4	W	Forward	ACCTCTCACCCACCACTCAC
		Reverse	AGCTCCCACCTGTCTCTCAA
OBSCN (var 1)	C	Forward	TACAAGGACGGGAAGAAACTG
		Reverse	TACCAGACATTCAACAACAGCAC
OBSCN (var 2)	C	Forward	CTTGGTGAGTTGCTTCATTGG
		Reverse	CCCTGAGAGAGCCTGTGAGA
OBSCN	J	Forward	GTGGGTTTATGTCTCTCATCAGC
		Reverse	CTTTCTGACTTTGTGAACTGTGG
OBSCN	U	Forward	GGTTCTAAAGGCGGTGGTCT
		Reverse	AGGAGGAGGTGGGAAGAGAAA
TNXB	2018-A	Forward	CATCAGTCTCCATGTCGCAAAAC
		Reverse	ATCTCCTCTTCCTGCTTTCCC
TNXB	G	Forward	GAGAAAGCAACATCTTCTAGGGC
		Reverse	ACCTGGCATCCTCTCTATATCTC
TNXB	S	Forward	GGACTATGAAGGAGTCAAACTGG
		Reverse	TTTTACATCAAAACCATCATGCGG
